# An Integrated Bioinformatics Analysis Reveals Divergent Evolutionary Pattern of Oil Biosynthesis in High- and Low-Oil Plants

**DOI:** 10.1371/journal.pone.0154882

**Published:** 2016-05-09

**Authors:** Li Zhang, Shi-Bo Wang, Qi-Gang Li, Jian Song, Yu-Qi Hao, Ling Zhou, Huan-Quan Zheng, Jim M. Dunwell, Yuan-Ming Zhang

**Affiliations:** 1 State Key Laboratory of Crop Genetics and Germplasm Enhancement, Nanjing Agricultural University, Nanjing 210095, People’s Republic of China; 2 Statistical Genomics Laboratory, College of Plant Science and Technology, Huazhong Agricultural University, Wuhan 430070, People’s Republic of China; 3 State Key Laboratory of Genetic Resources and Evolution, Kunming Institute of Zoology, Chinese Academy of Sciences, Kunming 650223, People’s Republic of China; 4 Institute of Biotechnology, Jiangsu Academy of Agricultural Science, Nanjing 210014, People’s Republic of China; 5 Department of Biology, McGill University, Montreal, Quebec H3A 1B1, Canada; 6 School of Agriculture, Policy and Development, University of Reading, Reading RG6 6AS, United Kingdom; INRA, FRANCE

## Abstract

Seed oils provide a renewable source of food, biofuel and industrial raw materials that is important for humans. Although many genes and pathways for acyl-lipid metabolism have been identified, little is known about whether there is a specific mechanism for high-oil content in high-oil plants. Based on the distinct differences in seed oil content between four high-oil dicots (20~50%) and three low-oil grasses (<3%), comparative genome, transcriptome and differential expression analyses were used to investigate this mechanism. Among 4,051 dicot-specific soybean genes identified from 252,443 genes in the seven species, 54 genes were shown to directly participate in acyl-lipid metabolism, and 93 genes were found to be associated with acyl-lipid metabolism. Among the 93 dicot-specific genes, 42 and 27 genes, including CBM20-like *SBDs* and *GPT2*, participate in carbohydrate degradation and transport, respectively. 40 genes highly up-regulated during seed oil rapid accumulation period are mainly involved in initial fatty acid synthesis, triacylglyceride assembly and oil-body formation, for example, *ACCase*, *PP*, *DGAT1*, *PDAT1*, *OLEs* and *STEROs*, which were also found to be differentially expressed between high- and low-oil soybean accessions. Phylogenetic analysis revealed distinct differences of oleosin in patterns of gene duplication and loss between high-oil dicots and low-oil grasses. In addition, seed-specific *GmGRF5*, *ABI5* and *GmTZF4* were predicted to be candidate regulators in seed oil accumulation. This study facilitates future research on lipid biosynthesis and potential genetic improvement of seed oil content.

## Introduction

The angiosperms are the most diverse group of land plants with the number of species in the range of 250,000 to 400,000 [1]. They have dramatic differences not only in organ morphology (leaves, flowers, seeds, roots, and vascular tissues) but also in the chemical composition of the seed [2]. Interestingly, oil plants, such as peanut, sesame and soybean, are generally rich in oil of their seeds, while most cereals like rice, wheat and sorghum specifically accumulate starch and have a relatively low oil content in the seed. Since the formation of seed oil and starch is both dependent on the supply of photosynthetic carbon [3, 4], it is likely that there are divergent mechanisms in the evolution of these high- and low-oil plants to regulate the partitioning of carbon between oil and other storage products. Previous studies have demonstrated that increasing the carbon flow to lipid biosynthesis can significantly increase the seed oils [4–6]. However, the mechanism by which more carbohydrates flow to *de novo* fatty acid (FA) synthesis in high-oil content plants is unclear.

Seed oil is not only the major source of carbon and energy for germination and seedling growth but also provides humans with renewable sources of food, biofuel and industrial raw materials. Up to now acyl-lipid metabolism in Arabidopsis has been well studied and more than 600 genes have been predicted to encode the enzymes and regulatory factors associated with this process [7, 8]. Among these predicted genes, some have been shown to be associated with changes in seed oil accumulation [6]. The over-expression of many individual key enzymes altered seed oil content in various plants. For example, ACCase in potato [9], *Brassica napus* [10] and *Escherichia coli* [11]; glycerol-3-phosphate dehydrogenase (GPDH) in *B*. *napus* [12]; glycerol-3-phosphate acyltransferase (GPAT) in *Arabidopsis* [13]; 2-lysophosphatidic acid acyltransferase (LPAAT) in *Arabidopsis* [14]; and acyl-CoA: diacylglycerol acytransferase (DGAT) in *Arabidopsis* [15, 16], *Glycine max* [17], *B*. *napus* [18] and maize [19]. In addition to these key enzymes that participate in lipid synthesis, the expression of transcription factors (TFs) directly or indirectly regulating genes involved in carbohydrate and lipid metabolism can also evidently change seed oil content; such TFs include *WRINKLED1* (*WRI1*) [20], *LEAFY COTYLEDON1* (*LEC1*) [21, 22], *LEAFY COTYLEDON2* (*LEC2*) [23], *FUSCA3* (*FUS3*) [24], *GmbZIP123* [25], *GmMYB73* [26] and *ABSCISIC ACID INSENSITIVE3* (*ABI3*) [27, 28]. However, each of the above studies only focused on a single enzyme or TF involved in lipid metabolism. In reality, seed oil content is affected by multiple genes [29, 30] or multiple reactions [31]. For example, specific combination of expression of *WRI1*, *DGAT* and triacylglycerol lipase *SUGAR-DEPENDENT1* resulted in a higher percentage seed oil content than that obtained by manipulation of each gene individually [32]. More importantly, the seed oil content is influenced by multiple metabolic pathways, such as sucrose catabolism, glycolysis, pentose phosphate pathway, and related pathways which rely on the supply of carbon [33–38]. Therefore, it is necessary to consider the involvement of multiple genes and the interaction of multiple related pathways in order to understand the mechanism of high-oil content in high-oil plants. The recent sequencing of many plant genomes has provided an opportunity to investigate this mechanism using comparative genome and transcriptome analyses.

Analysis of genome-wide differential gene expression between developmental stages, and between sub-species (like 输入文字或网址, 即可翻译 cultivated and wild forms) could provide insights into biological pathways and molecular mechanisms that regulate seed development and nutrient accumulation. Seed development is also an important part of the reproductive (R) process in flowering plants. In soybean, this reproductive process is divided into eight stages from flowering (R1) to full maturity (R8) [39]. During the R4 to R7 stages, importantly, seeds grow rapidly, accumulating nutrients, lipids and storage proteins.

In this study, all the genes from three low-oil grasses (*Sorghum bicolor*, *Setaria italica*, and *Oryza sativa*) and four high-oil dicots (*Glycine max*, *Gossypium raimondii*, *Ricinus communis*, and *Arabidopsis thaliana*) were clustered in order to obtain a list of high-oil dicot-specific genes. A gene ontology (GO) enrichment analysis and a pathway level co-expression (PLC) network analysis were then conducted to identify genes or TFs that are likely to be associated with oil accumulation. Analyses of gene expression during the various stages of seed development in soybean and of RNA sequencing (RNA-seq) differential expression between high- and low-oil content soybean accessions were performed to identify differentially expressed genes (DEGs) in the core pathways associated with the deposition of seed oil. These results were used to further investigate how evolutionary divergence contributes to differences in seed oil content between the two kinds of plants and to discover new genes associated with the seed oil differences.

## Results

### Identification and GO enrichment analysis of dicot-specific genes

In this study, OrthoMCL was applied to construct potential orthologous groups (OGs) of proteins across four high-oil dicots (seed oil content: 20–50%) and three low-oil grasses (<3%) (S1 Table), because it can group both orthologs and paralogs over multiple eukaryotic taxa by using a Markov Cluster algorithm (MCL) [40]. As a result, all the 252,443 genes from the above seven species were clustered into 29,095 OGs (S1 Dataset). Among these gene families, only 1,534 (5.27%) OGs appear to be specific to the high-oil dicot lineage and are defined as high-oil dicot-specific clusters since all the genes in these OGs come from all the four rosid species but not from any grasses (S1 Dataset). Note that OrthoMCL clusters proteins based on overall conservation but not on individual protein domains. Thus, high-oil dicot-specific OGs in this study contain families specific in high-oil dicots or families with much low similarities between dicots and grasses. Among the 1,534 high-oil dicot-specific clusters, 4,051, 2,758, 1,731 and 2,152 genes were found in *G*. *max*, *G*. *raimondii*, *R*. *communis*, and *A*. *thaliana*, respectively.

To understand the functions of these high-oil dicot-specific genes, a GO enrichment analysis was conducted for 4,051 soybean genes compared with all the annotated genes. 392 GO terms for biological processes, molecular functions and cellular components were identified; and these were distributed in 77 GO slim terms (S2 Dataset).

Among the 43 GO slims for biological processes, some were involved in metabolic pathways (S2 Dataset), such as biosynthetic process, carbohydrate metabolic process, catabolic process, generation of precursor metabolites and energy, lipid metabolic process, metabolic process, protein metabolic process and secondary metabolic process. In addition, slim GO:0006810 (transport) was involved in transport of many intermediates of carbohydrate degradation and glycolytic pathways, which includes triose phosphate transmembrane transport, phosphoenolpyruvate transport, acylglycerol transport, glucose-6-phosphate transport, phosphoglycerate transport, hexose phosphate transport, regulation of intracellular transport and triose phosphate transport.

### Expression patterns of dicot-specific genes during seed development

**Fig 1:**
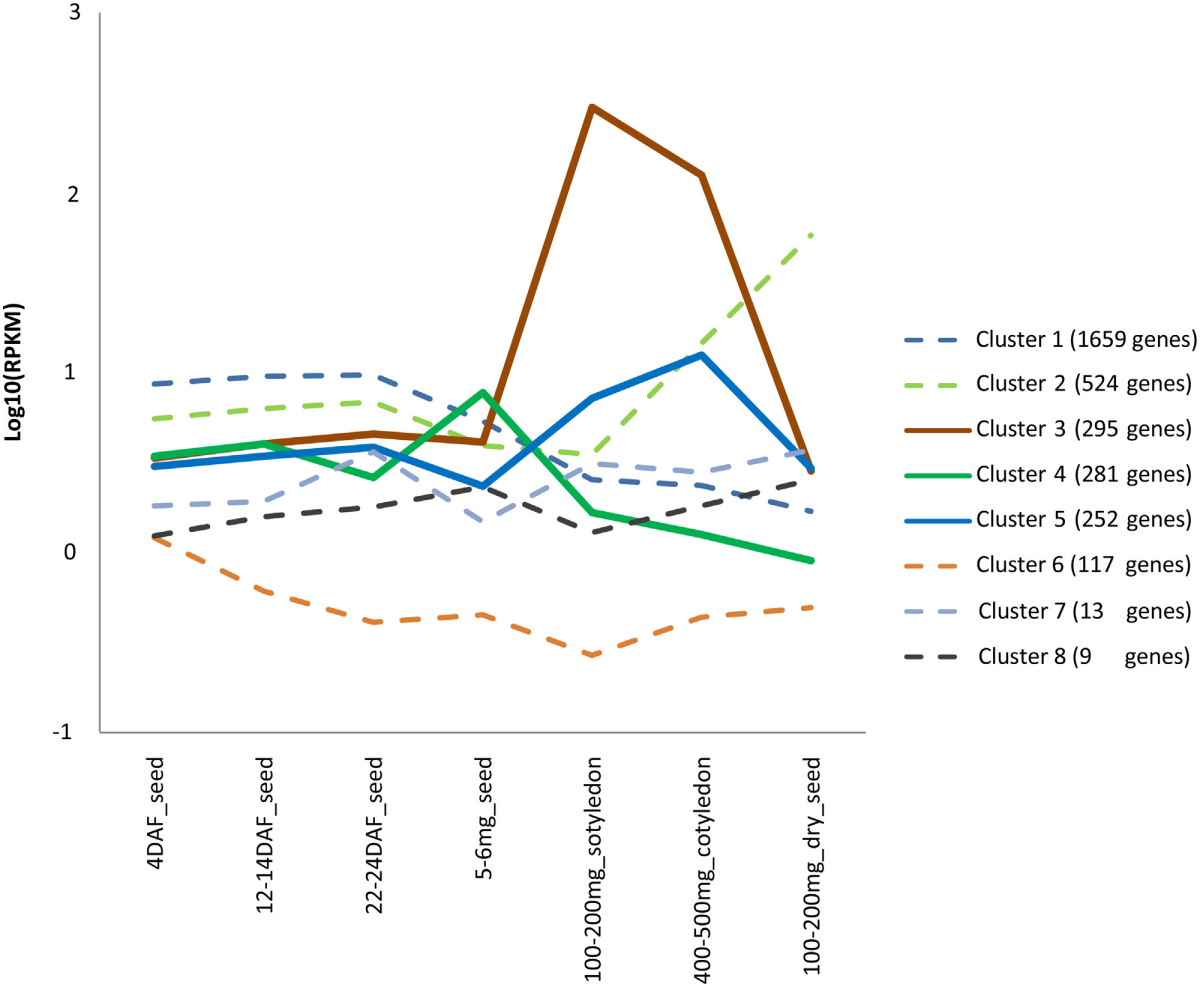
Average expression profile (*y* axis) for each cluster of dicot-specific genes at seven stages of soybean seed development (*x* axis). RPKM: reads per kilobase of gene model per million mapped reads; DAF: days after flowering. The dataset was downloaded from http://www.ncbi.nlm.nih.gov/geo/query/acc.cgi?acc=GSE42871.

The transcriptomic data for soybean seeds at seven stages of development [41] were used to analyze the expression patterns of 4,051 dicot-specific genes. 3,155 (77.88%) genes were expressed in developing seeds across two biological replicates. Among these 3,155 genes, 3,150 (99.84%) were grouped into eight clusters based on Pearson’s correlation coefficients, implemented by MCL [42]. All the eight clusters are shown in Fig 1 and the genes in each cluster are listed in S1 Dataset.

### Identification of genes related to seed oil content in soybean

#### Identification of high-oil dicot-specific genes directly participating in acyl-lipid metabolism

Compared with acyl-lipid metabolism genes in *Arabidopsis* [7, 8], 1,123 orthologous genes were identified in soybean (S1 Dataset). Among these 1,123 genes, 54 were high-oil dicot-specific genes and distributed in the above eight clusters (S2 Table). In particular, almost all the genes coding key enzymes of fatty acid synthesis like Biotin Carboxylase (BC), Biotin Carboxyl Carrier Protein 2 (BCCP2), Carboxyltransferase alpha subunit (α-CT), WRI1, and FUS3 were in cluster 3.

#### Dicot-specific lipid-metabolism-related genes predicted from PLC analysis

Genes in the clusters 3 to 5 show highest expression levels at one of the stages from 5–6 mg to 400–500 mg, which tend to occur between R4 and R7. During these stages, lipids are rapidly accumulated in the seed [39]. To ensure prediction accuracy, these genes in clusters 3 to 5 were selected for further analysis. This is because that the genes in cluster 2 show highest expression at the dry whole seed stage and the ones in the remaining clusters are down-regulated during seed development. In the clusters 3 to 5, there were 828 genes (S1 Dataset), among which 23 genes have been determined to participate in the above lipid synthesis (S2 Table). GO enrichment analysis for the remaining 805 genes assigned 207 (25.71%) genes to the biological processes that may be associated with carbohydrate and lipid metabolic pathways (S3 Dataset and S3 Table), such as carbohydrate metabolic process, lipid metabolic process, transport, signal transduction, and catabolic process.

To further identify and prioritize novel candidate acyl-lipid metabolism members in these three clusters, we conducted a pathway-level coexpression (PLC) network analysis [43–46] between the above 207 genes and 1,123 genes in acyl-lipid metabolism. The results show that 93 dicot-specific genes are candidate genes involved in acyl-lipid metabolism pathways (Table 1, S4 Table and S5 Dataset).

**Table 1:** Dicot-specific genes associated with acyl-lipid metabolism.

GO slims	Dicot-specific genes	Pathway annotation	Pfam annotation	Enzymes/Proteins	Reference
GO:0005975; Carbohydrate metabolic process	*Glyma0165s00200*, *Glyma01g28520*, *Glyma03g08860*		PF00686	*CBM20-like* starch binding domian	Marchler-Bauer et al. [67]; Southall et al. [70]; Rodriguez-Sanoja et al. [69]; Christiansen et al. [68]
	***Glyma09g04330***, *Glyma15g15360*	PWY-6902; chitin degradation II	PF00182	chitinase	
	*Glyma01g33440*, *Glyma03g03400*, *Glyma03g03460*, *Glyma06g47690*, *Glyma06g47690*, *Glyma12g00700*	PWY-1081; homogalacturonan degradation	PF01095, PF04043	pectinesterase	
	***Glyma04g02660*, *Glyma06g02690***, *Glyma14g40400*, ***Glyma17g37750*, *Glyma17g37760***		PF02704	Gibberellin-regulated family protein	Aubert et al. [97]; Chen et al. [98]
	*Glyma01g37150*, ***Glyma11g08120***, *Glyma15g08960*, *Glyma05g36680*, *Glyma14g40110*, ***Glyma02g34870***, *Glyma03g36250*, ***Glyma10g10530***, ***Glyma19g38900***, *Glyma02g36150*, *Glyma10g08740*, *Glyma08g16880*, *Glyma09g01520*, *Glyma17g36200*, *Glyma14g08970*, *Glyma10g16100*, *Glyma07g05970*, *Glyma04g40630*, *Glyma06g14160*, *Glyma05g06410*, *Glyma19g07830*, *Glyma18g00560*, *Glyma02g47500*, ***Glyma14g01260***, *Glyma17g09260*		S4 Table		
GO:0006810; Transport	*Glyma07g38830*, *Glyma13g27680*, ***Glyma15g11270***		PF03151, PF00892	*GPT2*	Kammerer et al. [71]; Niewiadomski et al. [74]; Andriotis et al. [73]; Kunz et al. [75]; Bourgis et al. [77]
	*Glyma18g08740*		PF03151	triosephosphate translocator subfamily protein	
	*Glyma12g13070*, ***Glyma08g02510***, *Glyma03g05880*, *Glyma15g17310*, *Glyma06g40980*, *Glyma07g12460*, *Glyma09g06260*, *Glyma12g34020*, *Glyma18g14810*, *Glyma09g04870*, ***Glyma15g15990***, *Glyma04g06230*, *Glyma03g33150*, *Glyma17g09640*, ***Glyma05g37050***, *Glyma09g08410*, *Glyma12g33880*, *Glyma19g34820*, *Glyma03g33660*, *Glyma06g22240*, *Glyma14g33680*, *Glyma20g23120*, *Glyma17g09260*		S4 Table		
GO:0006629; Lipid metabolic process	*Glyma02g45680*, *Glyma14g03130*		PF00067	cytochrome P450, family 718	Pinot and Beisson [99]; Sun et al. [100]
	*Glyma18g45250*	PWY-2761; glyceollin biosynthesis I	PF01370	pterocarpin synthase	Kim et al. [101]
	***Glyma02g46220***, *Glyma14g02510*		S4 Table		
GO:0007165; Signal transduction	*Glyma09g07090*, ***Glyma15g18380***, *Glyma17g06290*		PF00320	GATA type zinc finger transcription factor family protein	Bi et al. [96]; Velmurugan et al. [95]
	*Glyma09g39570*, *Glyma10g12130*	PWY-5035; gibberellin biosynthesis III	PF03171	gibberellin 3β-dioxygenase	Chen et al. [98]
	*Glyma12g32910*, ***Glyma13g39340***, *Glyma02g47500*, *Glyma14g01260*, *Glyma13g29070*, *Glyma15g09980*		S4 Table		
GO:0019538; Protein metabolic process	*Glyma05g23620*, *Glyma17g16690*, *Glyma01g03580*, *Glyma06g10110*, *Glyma08g39290*, *Glyma17g12200*, *Glyma18g19720*		S4 Table		

High-oil dicot-specific genes were predicted by PLC analysis. 17 genes with bold type were also differentially expressed in the high- and low-oil soybean seeds at the 0.01 significant level. All the information containing both annotation and P-value in differential expression analysis for each gene is given in S4 Table.

#### Expression patterns of genes encoding core lipid synthetic enzymes

**Fig 2:**
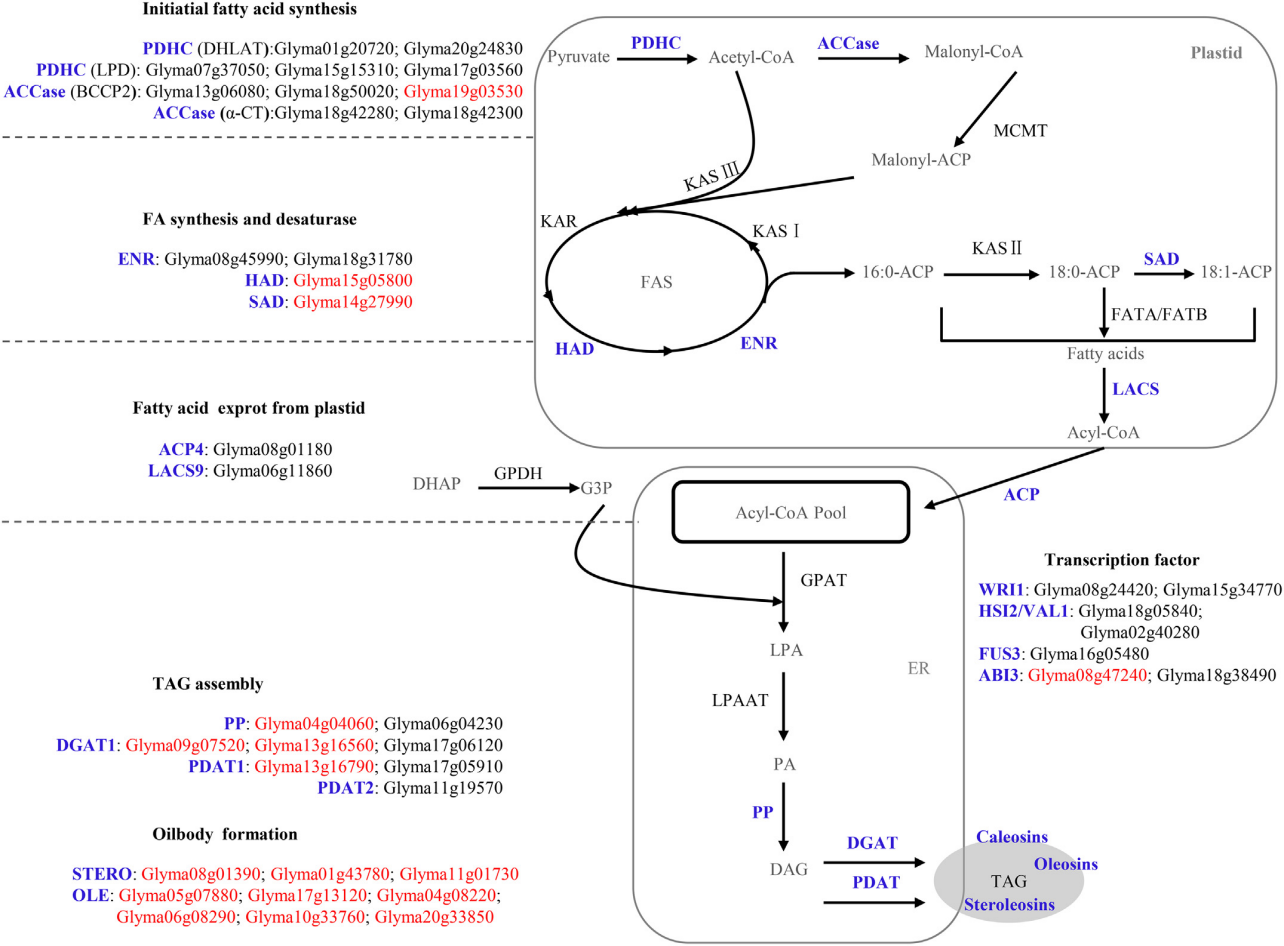
The distribution of 40 highly up-regulated genes and the respective DEGs in the framework of acyl-lipid metabolism. Enzymes with blue color are highly up-regulated and 40 genes coding them are listed. Among these 40 highly up-regulated genes, DEGs between the high- and low-oil soybean accessions are highlighted with red color. ABI, ABSCISIC ACID INSENSITIVE; ACP, acyl carrier protein; BCCP, biotin carboxyl carrier protein; DGAT, acyl-CoA: diacylglycerol acytransferase; DHAP, Dihydroxyacetone phosphate; DHLAT, dihydrolipoamide acetyltransferase; ENR, enoyl-ACP reductase; ER, endoplasmic reticulum; FAS, fatty acid synthase; FATA(B), fatty acylthioesteraseA(B); FUS, FUSCA; G3P, glycerol-3-phosphate; GPAT, glycerol-3-phosphate acyltransferase; GPDH, glycerol-3-phosphate dehydrogenase; HAD, hydroxyacyl-ACP dehydrase; KAR, ketoacyl-ACP reductase; KAS, ketoacyl-ACP synthase; LACS, long-chainacyl-CoA synthetase; LPD, dihydrolipoamide dehydrogenase; MCMT, malonyl-CoA:ACP malonyltransferase; OLE, oleosins; PDAT, phospholipid:diacylglycerolacyl transferase; PDH, pyruvatede hydrogenase; PP, phosphatidate phosphatase; SAD, stearoyl-ACP desaturase; STERO, steroleosin; TAG, triacylglycerol; WRI, WRINKLED.

In soybean, the core pathways for accumulating seed oil operate through FA synthesis and the export of FAs from the plastid followed by triacylglycerol synthesis, and oil body formation, which included 156 genes (S1 Dataset). Among these genes, 113 (72.44%) had higher expression level than the average in at least one of the stages from 5–6 mg to 400–500 mg (S4 Dataset), indicating that most key genes for lipid synthesis were up-regulated during oil accumulation in *G*. *max*. This phenomenon was similar to that in *Arabidopsis* [47]. If the criterion used was twice average level, 40 (25.6%) genes were identified (S4 Dataset). These 40 genes were largely distributed in the initiation of FA synthesis, triacylglycerol synthesis, and oil-body formation (Fig 2).

**Fig 3:**
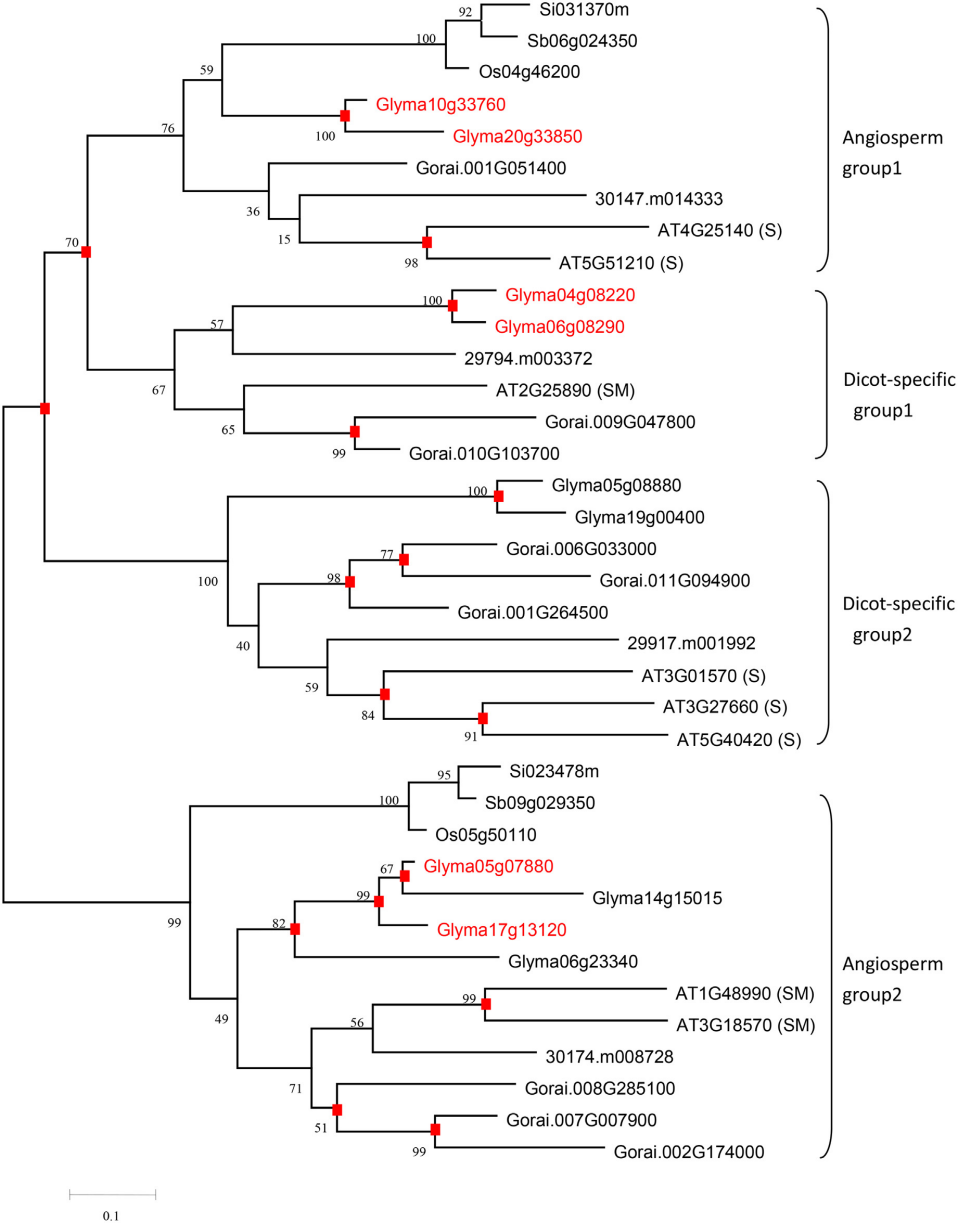
Phylogenetic tree of oil body protein OLE. The phylogenetic tree was constructed using Neighbor-Joining method. The numbers on the branches represent the bootstrap support, and square boxes indicate duplication events. S indicates genes expressed only in maturing seed and SM in both maturing seeds and florets. The highly up-regulated genes in Fig 2 are highlighted in red color.

Fourteen of the above 40 genes were observed in FA synthesis, and 10 of the 14 genes were involved in the initiation of FA synthesis. Among the 10 genes, five encoded two subunits of the plastidial pyruvate dehydrogenase complex (PDHC) that promotes pyruvate decarboxylation to acetyl-CoA and is the key enzyme linking carbohydrate metabolism to FA synthesis [4]; and the others encoded two subunits of ACCase. Of these 40 genes, two genes, those encoding ACP4 and LACS9, were involved in FA transportation. Eight of the above 40 genes were involved in TAG assembly; this group included 2, 3, 2, and 1 genes respectively coding phosphatidate phosphatase (PP), DGAT1, phospholipid: diacylglycerol acyltransferase (PDAT1), and PDAT2; these enzymes catalyze the consecutive steps after the second acylation of glycerol-3-phosphate [15, 19, 48]. Three and six of the above 40 genes, respectively encoding steroleosins (STEROs) and oleosins (OLEs), were observed in oil-body formation, and they were up-regulated, mostly by some hundredfold, during stages from 5–6 mg to 400–500 mg in seeds (S4 Dataset). The remaining genes in this group of 40 genes encoded the TFs WRI1, VAL1, FUS3, and ABI3. Kim et al. [49] divided Arabidopsis oleosin genes into three groups on the basis of their tissue-specific expression. In this study, all the genes in the S type (genes expressed only in maturing seed (siliques)) and SM type (genes expressed in both maturing seeds and florets (microspores)) were used to conduct a phylogenetic analysis (Fig 3). The results showed distinct differences of OLEs in patterns of gene duplication and loss between high-oil dicots and low-oil grasses. Genes coding oleosins were preferentially retained in high-oil dicots. In two angiosperm groups, there was only one copy in each grass species, while there were many duplicates in dicots. Remarkably, two high-oil dicot-specific groups were identified, which were resulted from gene loss in grasses after whole genome duplication of ancestor angiosperm. In dicot-specific group 1, *Glyma04g08220* and *Glyma06g08290* were up-regulated by a thousand-fold during stages from 5–6 mg to 400–500 mg (S4 Dataset) and may play an important role in soybean seed oil accumulation. In dicot-specific group 2, *At3G01570*, *At3G27660* and *At5G40420* in Arabidopsis were shown to be S type genes [49]. Meanwhile, *Glyma05g08880* and *Glyma19g00400* in this group were highly up-regulated in hundred-fold in the dry whole seed (S4 Dataset), indicating that this dicot-specific group may perform some specific functions in seed development. Furthermore, highly up-regulated and abundant OLEs probably play major roles in oil accumulation and/or oil body development, and are associated with high-oil content in seeds [50, 51].

Interestingly, *OLEs*, *STEROs* and *ABI3* genes are all significantly over-expressed and co-expressed with each other (S1 Fig and S4 Dataset). Since *OLEs* were regulated by *ABI3* [28], *ABI3* might also up-regulate the expression of *STEROs*. To improve seed oil content in molecular breeding, it may be beneficial to increase the expression of *OLEs*, *STEROs* or *ABI3* genes.

#### Identification of candidate TF genes related to seed oil accumulation

Several important transcription factors (TFs) have been found to participate in the regulation of seed oil accumulation. These TFs are distributed in the TF families like AP2, basic leucine zipper (bZIP), B3, NF-YB and Dof [21, 22, 24–27, 52, 53]. In soybean, there were 3,714 genes coding TFs [54], and 19 of these were found to be known lipid-related TFs (S5 Dataset). To identify additional potential TFs related to seed oil accumulation, we conducted a PLC network analysis between all the TFs in soybean and the above 156 genes coding core lipid synthetic enzymes. As a result, 327 TFs were found to be co-expressed with at least two genes coding core lipid synthetic enzymes (P-value<1e-4) (S5 Dataset), and were classified into 42 gene families. Among the 327 TFs, 67 were distributed in high-oil dicot-specific clusters (S5 Dataset), and 70 had expression levels greater than twice the average level in at least one of stages from 5–6 mg to 400–500 mg. These 70 TFs were compared with the 69 seed-specific TFs described by Song et al. [25]. Seven genes were found to be seed-specific, respectively coding tandem CCCH zinc finger protein 4 (*GmTZF4*; *Glyma12g13300*, *Glyma06g44440*, *Glyma12g33320*), growth-regulating factor 5 (*GmGRF5*; *Glyma07g04290*), ABI5 (*Glyma10g08370*), ABI3 (*Glyma18g38490*) and a sequence-specific DNA binding TF (*Glyma03g34730*) (Table 2). Since these seven seed-specific TFs were not only highly up-regulated during the rapid oil accumulation phase of seed development but also co-expressed with key enzymes of lipid synthesis genes, we deduced that they possibly play roles in regulating seed oil accumulation. It should be noted that ABI3 has been confirmed to be a regulator in oleosin gene expression [17, 28].

**Table 2:** Pathway level co-expression analysis between seven seed-specific transcript factors and genes in the key pathways of lipid synthesis.

Transcript factor	Subfamily	P-value in differential expression analysis		Pathway-level co-expression analysis				Arabidopsis homolog of soybean transcript factor
		Group H-L1	Group H-L2	Gene	Abbreviation	r	P-value	
*Glyma12g13300* (*GmTZF4*)	C3H	3.73e-34	3.10e-09	*Glyma18g05840*	*VAL*	0.9728	5.87e-05	*At1g03790* (*AtTZF4*; *SOM*)
				*Glyma04g08220*	*OLE*	0.9930	1.05e-06	
				*Glyma06g08290*	*OLE*	0.9891	3.87e-06	
				*Glyma09g07520*	*DGAT1*	0.9683	9.26e-05	
				*Glyma08g01390*	*STERO*	0.9997	8.20e-11	
				*Glyma07g32850*	*SAD*	0.9898	3.16e-06	
*Glyma06g44440* (*GmTZF4*)	C3H	6.21e-31	1.29e-09	*Glyma19g03530*	*BCCP2*	0.9757	4.22e-05	*At1g03790* (*AtTZF4*; *SOM*)
				*Glyma04g08220*	*OLE*	0.9855	9.10e-06	
				*Glyma09g07520*	*DGAT1*	0.9929	1.08e-06	
				*Glyma11g19570*	*PDAT2*	0.9717	6.62e-05	
				*Glyma08g01390*	*STERO*	0.9871	6.39e-06	
				*Glyma07g32850*	*SAD*	0.9768	3.68e-05	
*Glyma12g33320* (*GmTZF4*)	C3H	8.79e-37	1.14e-07	*Glyma03g29600*	*LPAAT*	-0.9759	4.08e-05	*At1g03790* (*AtTZF4*; *SOM*)
				*Glyma19g32420*	*LPAAT*	-0.9705	7.43e-05	
				*Glyma18g38490*	*ABI3*	0.9904	2.66e-06	
*Glyma07g04290* (*GmGRF5*)	GRF	2.20e-13	3.15e-08	*Glyma09g07520*	*DGAT1*	0.9915	1.84e-06	*At3g13960* (*AtGRF5*)
				*Glyma11g19570*	*PDAT2*	0.9912	2.06e-06	
*Glyma03g34730*	Trihelix	1.83e-17	2.58e-15	*Glyma13g06080*	*BCCP2*	0.9715	6.76e-05	
				*Glyma19g03530*	*BCCP2*	0.9798	2.43e-05	
				*Glyma09g07520*	*DGAT1*	0.9940	6.63e-07	
				*Glyma11g19570*	*PDAT2*	0.9934	8.84e-07	
*Glyma10g08370* (*ABI5*)	bZIP	4.66e-21	5.97e-09	*Glyma03g29600*	*LPAAT*	-0.9737	5.30e-05	*At2g36270* (*ABI5*)
				*Glyma19g32420*	*LPAAT*	-0.9831	1.43e-05	
				*Glyma01g43780*	*STERO*	0.9751	4.49e-05	
				*Glyma08g47240*	*ABI3*	0.9692	8.49e-05	
				*Glyma18g38490*	*ABI3*	0.9927	1.18e-06	
*Glyma18g38490* (*ABI3*)	B3	9.51e-15	1.26e-01	*Glyma03g29600*	*LPAAT*	-0.9855	8.98e-06	*AT3G24650* (*ABI3*)
				*Glyma19g32420*	*LPAAT*	-0.9787	2.85e-05	

Co-expression network analysis was conducted between seven seed-specific TFs and genes involved in the core lipid synthesis pathways at the 1e-04 level. Group H-L1: HanDou 5 (high-oil) and ZYD4364 (low-oil); Group H-L2: HanDou 5 and Y117249 (low-oil).

We investigated co-expression networks of the above 19 known lipid-related TFs with 156 genes coding core lipid synthetic enzymes. As a result, 14 lipid-related TFs were co-expressed with more than 10 lipid synthesis genes (P-value<0.05) (S5 Dataset), such as two genes (*Glyma15g34770* and *Glyma08g24420*) coding WRI1 were found to be co-expressed with 19 lipid synthesis genes (Table 3). Among the 19 lipid synthesis genes, genes coding BCCP2, BC, ENR and α-PDH have been verified to be regulated by *WRI1* in *Arabidopsis* [53, 20, 23], and 18 genes have an AW-box in their promoter (S2 Fig) that has been shown to be a direct target of WRI1 in *Arabidopsis* [53]. Note that *WRI1* has a high co-expression relationship with *Glyma06g11860* (*LACS9*; r = 0.9801, P-value = 2.32e-5), and *LACS9* is found to impact the biosynthesis of seed storage lipids in *Arabidopsis* [55], and is considered as the major LACS isoform involved in plastidial FA export for TAG formation [56]. We surmised that *WRI1* up-regulated *LACS9* and this regulation could lead to an increased export of FAs to the endoplasmic reticulum (ER), and a subsequent increase in the rate of FA synthesis and triacylglycerol synthesis.

**Table 3:** Soybean genes co-expressed with *GmWRI1*.

Gene			Co-expression analysis with *Glyma08g24420*		Co-expression analysis with *Glyma15g34770*		Similar result in Arabidopsis	
ID	Abbreviation	No. of AW-boxes in promoter	r	P-value	r	P-value	Homologous gene	Reference
*Glyma13g06080*	*BCCP2*	4	0.8105	1.53e-02	0.8491	8.24e-03	*At5g15530*	Baud et al. [20]; Maeo et al. [53]; Fukuda et al. [110]
*Glyma18g50020*	*BCCP2*	4	0.8844	3.93e-03	0.8771	4.66e-03	*At5g15530*	
*Glyma19g03530*	*BCCP2*	3	0.7445	3.36e-02	0.7842	2.17e-02	*At5g15530*	
*Glyma05g36450*	*BC*	1	0.8222	1.29e-02	0.7981	1.82e-02	*At5g35360*	Fukuda et al. [110]
*Glyma08g03120*	*BC*	1	0.9482	3.89e-04	0.9513	3.26e-04	*At5g35360*	
*Glyma07g37050*	*LPD*	3	0.8636	6.24e-03	0.8602	6.67e-03		
*Glyma15g15310*	*LPD*	1	0.8357	1.04e-02	0.8177	1.38e-02		
*Glyma17g03560*	*LPD*	3	0.8222	1.29e-02	0.8202	1.33e-02		
*Glyma08g45990*	*ENR*	2	0.8129	1.48e-02	0.8077	1.59e-02	*At2g05990*	Baud et al. [23]
*Glyma18g31780*	*ENR*	2	0.7291	3.90e-02	0.7354	3.68e-02	*At2g05990*	
*Glyma18g36130*	*FATA*	2	0.7311	3.83e-02	0.7522	3.11e-02		
*Glyma07g05550*	*α-PDH*	2	0.9671	1.03e-04	0.9646	1.28e-04	*At1g01090*	Baud et al. [23]
*Glyma16g02090*	*α-PDH*	2	0.9464	4.31e-04	0.9539	2.77e-04	*At1g01090*	
*Glyma09g07520*	*DGAT1*	3	0.7525	3.10e-02	0.7773	2.35e-02		
*Glyma06g11860*	*LACS9*	2	0.9801	2.32e-05	0.9875	5.81e-06		
*Glyma18g42280*	*α-CT*	1	0.7890	2.04e-02	0.7591	2.89e-02		
*Glyma18g42300*	*α-CT*	2	0.7972	1.84e-02	0.7664	2.67e-02		
*Glyma11g19570*	*PDAT2*	0	0.8025	1.71e-02	0.8144	1.45e-02		
*Glyma18g06500*	*MCMT*	2	0.8225	1.29e-02	0.8069	1.61e-02		

Two soybean genes (*Glyma08g24420* and *Glyma15g34770*) are homologous to *AtWRI1*, and all the genes co-expressed with the two soybean genes at the 0.05 level were listed in this table.

Interestingly, the expression of *WRI1* could increase seed oil content in both dicots and grasses, such as in *Arabidopsis* [20], oilseed rape [57], oil palm [58, 59] and maize [60]. However, distinct differences exist between dicots and grasses. These differences include low sequence similarity that results in dicot-specific and grass-specific clusters (S1 Dataset), different gene structures, and a low evolutionary rate in dicots (ω1 = 0.0861) as compared with that in grasses (ω0 = 0.2809) (S3 Fig). More importantly, *WRI1* is included in different regulatory networks in dicots and grasses [61]. In *Arabidopsis*, the expression of *WRI1* is up-regulated by *LEC1*, *LEC2* and *FUS3*, and *WRI1* is a direct target of *LEC2* [23], and possibly of *FUS3* [62]. In maize, however, no ortholog of *AtLEC2* was identified [60], and *WRI1* is able to regulate amino acid biosynthesis [63]. In addition, *LEC1* and *FUS3* in *Arabidopsis* have low sequence similarity compared with those in grasses.

### Differential expression analysis between high- and low-oil soybean accessions

A differential expression analysis to detect up-regulated genes in the high-oil materials may contribute to a better understanding of high-oil content mechanisms. In this study, RNA-seq differential expression analysis between high-oil cultivar Handou 5 (HD5; seed oil content: 22.3%) and two low oil wild soybeans ZYD4364 (11.9%) and Y117249 (12.5%) was conducted in the seeds at four developmental stages (15, 25, 35 and 55 days after flowering (DAF)). As a result, 8,356 DEGs between accessions HD5 and ZYD4364 (Group H-L1), and 5,551 DEGs between accessions HD5 and Y117249 (Group H-L2) were identified, and 3,997 common DEGs were observed (P<0.01).

**Fig 4:**
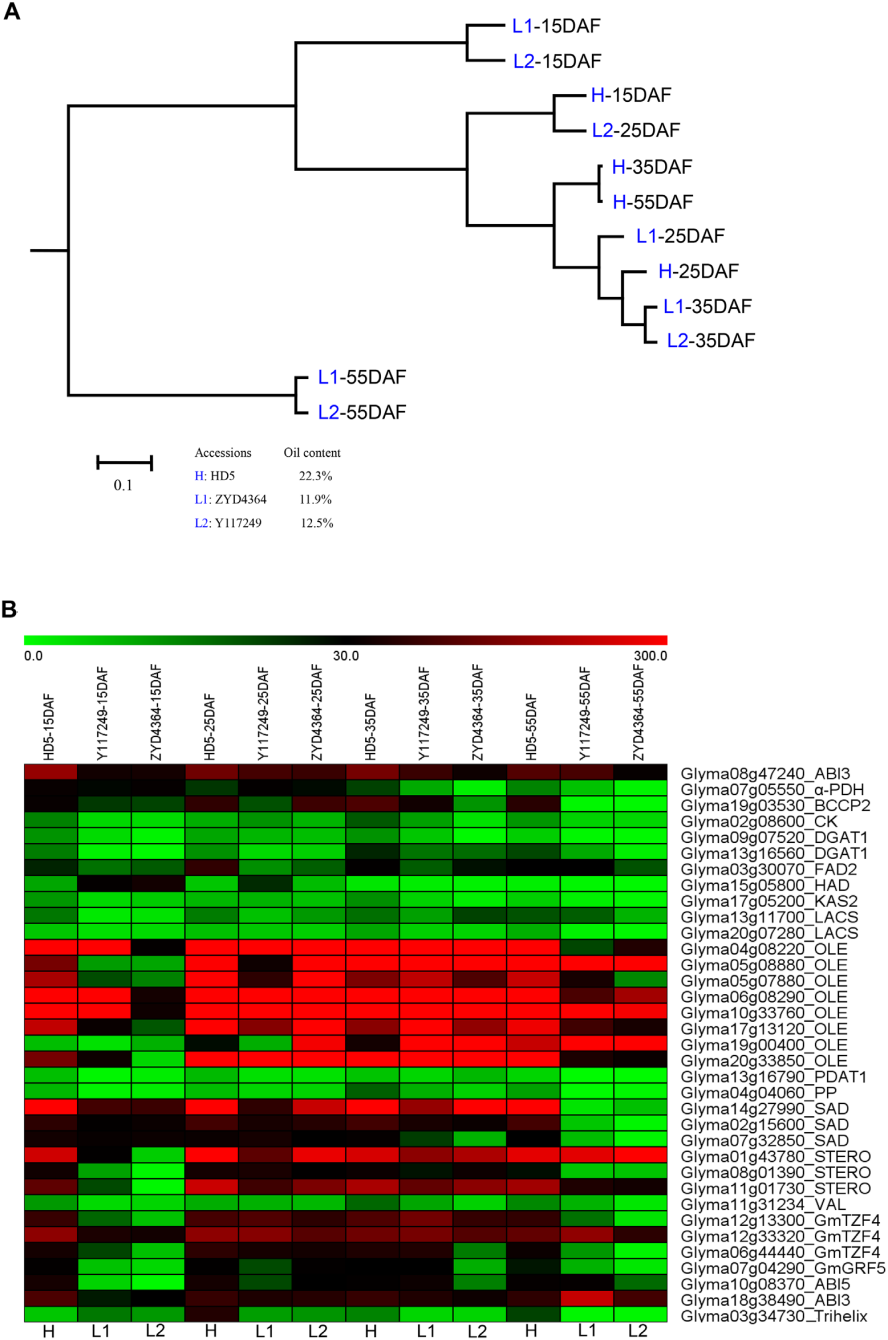
Expression profiles of acyl-lipid metabolism genes in high- and low-oil soybean seeds. A: Hierarchical clusters of the soybean seed samples using expression levels of 1,123 genes in acyl-lipid metabolism. 15DAF (15 days after flowering), 25DAF, 35DAF and 55DAF are four stages of seed development. B: Expression patterns of 28 differentially expressed genes in core lipid synthesis pathways. Seven seed-specific TFs are also shown. HD5 (high-oil content in seed, 22.3%), ZYD4364 (low-oil content in seed, 11.9%) and Y117249 (low-oil content in seed, 12.5%) indicate the sample codes.

Among 1,123 acyl-lipid metabolism genes, 77 were differentially expressed in the above two groups (H-L1 and H-L2), and 28 DEGs encoded core lipid synthetic enzymes (S2 Table and Fig 4B). We found that 28 DEGs in core lipid synthesis pathways were up-regulated in all the three accessions at stages 25 and 35 DAF, during which seed oil is rapidly accumulated (Fig 4B), indicating that they play vital roles in seed oil accumulation. Interestingly, at early (15 DAF) and late (55 DAF) stages, the 28 DEGs still expressed at a relative high level in high-oil accession but at a relative low level in both low-oil accessions (Fig 4B). This phenomenon was also observed in seven seed-specific lipid-related TFs. Cluster analysis of expression patterns for the 1,123 acyl-lipid metabolism genes clearly distinguished the 15 and 55 DAF from the other two stages, and the high-oil accession from the low oil accessions (Fig 4A), indicating that divergent expression patterns of acyl-lipid metabolism genes in the early and late seed development stages played roles in determining different oil content [64]. The above 28 DEGs in core lipid synthesis pathways were compared with the above 40 highly up-regulated genes. As a result, 17 common genes were found; these are shown in red in Fig 2, and encode BCCP2, HAD, SAD, PP, DGAT1, PDAT1, STERO, OLE, and ABI3. These common genes were not only up-regulated during the rapid oil accumulation phase of seed development but also differentially expressed in high- and low-oil accessions, indicating the importance of these key enzymes in determining seed oil content. Clearly, there must be a relationship between high expression pattern and key proteins. However, there are some minor exceptions; for example, *PDAT1* and *PDAT2* in *Arabidopsis* have no effects on seed oil content and TAG synthesis, respectively, although the two genes are highly expressed during seed development [65, 66].

Differential expression analysis was also used to validate the candidate lipid-related genes predicted by PLC analysis. As a result, 17 of the 93 dicot-specific lipid-related genes (Table 1 and S4 Table), and 61 of the 327 lipid-related TFs were found to be differentially expressed in the above two groups (H-L1 and H-L2) (S5 Table). More importantly, all the seven seed-specific TFs were also differentially expressed in high- and low-oil soybean accessions, except *Glyma18g38490* coding ABI3, which was differentially expressed only between accessions HD5 and ZYD4364 (Table 2).

## Discussion

### Carbohydrate degradation and transport

The *de novo* synthesis of FAs in plants has a close connection with carbohydrate metabolism and transportation of its metabolic intermediates [6]. Interestingly, of the 93 dicot-specific genes putatively related to lipid metabolism, 42 (45.16%) and 27 (29.03%) genes were included in carbohydrate metabolic process and transport, respectively (Table 1 and S4 Table).

The 42 genes associated with carbohydrate metabolism were classified into three sub-groups. The first one had three genes (*Glyma0165s00200*, *Glyma01g28520*, *Glyma03g08860*), encoding proteins with a conserved domain PF00686 (starch binding domain; SBD) (Table 1 and S4 Table), which is also known as the family 20 carbohydrate-binding module (CBM20) and is found in great many starch degrading enzymes including alpha-amylase, beta-amylase, glucoamylase, and cyclodextrin glucanotransferase [67]. SBD could mediate the attachment between starch-active enzymes and starch granules and might disrupt the structure of the starch surface, thereby enhancing the amylolytic rate [68–70]. Accordingly, these three CBM20-like proteins possibly promote the hydrolysis of starch by combining with some amylolytic enzymes or other related glycosidases. Although all the three CBM20-like genes were not differentially expressed at the 0.01 probability level, *Glyma01g28520* was differentially expressed at the 0.05 level (S4 Table).

The second sub-group includes the dicot-specific genes that participate in the degradation of other carbohydrates, such as homogalacturonan degradation (PWY-1081) and chitin degradation II (PWY-6902) (Table 1 and S4 Table). Note that *Glyma09g04330* in chitin degradation II was differentially expressed between high- and low-oil soybean accessions. These carbohydrates degradation genes might be involved in the increase in carbon sources during FA biosynthesis. The last sub-group comprised some dicot-specific genes with unknown functions (Table 1 and S4 Table).

Among the 27 dicot-specific genes in transport, four genes (*Glyma07g38830*, *Glyma13g27680*, *Glyma15g11270* and *Glyma18g08740*) belonged to *PF03151* (Triose-phosphate Transporter family). The first three genes were annotated as genes encoding the glucose 6-phosphate/phosphate translocator 2 (GPT2), which is located in the envelope of the plastids for the importation of Glc6P [71]. In non-green plastids, glucose 6-phosphate (Glc6P) can be used as a precursor for starch biosynthesis and FA synthesis and also as a substrate for the Oxidative Pentose Phosphate Pathway (OPPP) which supplies reducing power to drive FA synthesis [4, 72]. Although *AtGPT2* has no obvious effects on plant development [73, 74], its over-expression could increase the net import of Glc6P from cytosol to chloroplast and accelerate the accumulation of soluble sugars in *Arabidopsis* [75, 76]. Moreover, the expression of *GPT2* in oil palm increased notably during fruit ripening and was significantly higher than in date palm, a low oil content palm [77]. More importantly, *Glyma15g11270* coding GPT2 was also differentially expressed in high- and low-oil soybean accessions (Table 1 and S4 Table). In that case, the specific GPT2 in high-oil content dicots implies a strong funneling of carbon toward pyruvate in the plastid, a significant increase for FA synthesis and ultimately an increase in the seed oil content [77]. The remaining 23 transport genes had unclear functions (Table 1 and S4 Table).

### FA synthesis and regulation of seed oil accumulation

#### Functions of dicot-specific genes encoding key enzymes of FA synthesis

Among the 23 dicot-specific genes encoding key enzymes of FA synthesis in clusters 3 to 5 (S2 Table), 10 are involved in FA synthesis, i.e., *Glyma05g36450* and *Glyma08g03120* encode Biotin Carboxylase (*BC*); *Glyma13g06080*, *Glyma18g50020* and *Glyma19g03530* encode Biotin Carboxyl Carrier Protein 2 (*BCCP2*); *Glyma18g42280* and *Glyma18g42300* encode Carboxyltransferase alpha subunit (*α-CT*); *Glyma15g34770* and *Glyma08g24420* belong to the *AP2/EREBP* family and encode soybean *WRI1*; and *Glyma16g05480* encodes the FUSCA3 (FUS3). All these genes except the two genes encoding BC were highly up-regulated during the rapid oil accumulation phase of seed development. *Glyma19g03530* encoding BCCP2 was up-regulated in high-oil soybean accessions compared with the two low-oil accessions (Fig 2).

*BC*, *BCCP* and *α-CT* are the subunits of heteromeric acetyl-CoA carboxylase (ACCase), which is the rate limiting enzyme of FA synthesis [78]. In dicots, there are two forms of Acetyl-CoA Carboxylase (ACCase), a heteromeric form in the plastid and a homomeric form in the cytosol. In grasses, however, ACCase in both the plastid and the cytosol is the homomeric type [79]. Previous studies have shown that ACCase is able to control the rate of carbon flow in plant leaves and its expression level is related to oil content in seed [9,11]. Specific heteromeric ACCase and its high expression level in high-oil dicots are probably key factors leading to the fact that oil content in high-oil dicots is significantly higher than that in grasses.

*WRI1* plays an important role in controlling the rate of carbon flow from carbohydrate metabolism to lipid synthesis, and is capable of affecting the seed oil accumulation by regulating a set of genes involved in lipid synthesis, glycolysis and photosynthesis [80, 57, 47]. Although *WRI1* could regulate seed oil accumulation in both grasses and high-oil dicots, different gene structures and divergent evolutionary rates between these two lineages were observed (S3 Fig). We deduced that different evolutionary mechanisms of *WRI1* in high-oil dicots and grasses might lead to different regulatory networks [61].

Although up-regulation of key enzymes in lipid synthesis like ACCase alone slightly increases oil content [11], its effect on increasing the oil content was much less than that by up-regulation of *WRI1* or other TFs [47, 59]. This partly indicates that oil accumulation is a complex biological process and increasing the expression of TFs may be an effective approach to significantly improve seed oil content.

#### Functions for up-regulated genes of FA synthesis in seed

In the initial period of FA synthesis, apart from five dicot-specific genes coding ACCase, five genes coding PDHC were also found to be highly up-regulated during the rapid oil accumulation phase of seed development (Fig 2). In several species, the expression of *PDHC* has been reported to be associated with seed oil content [81–83]. In this study, *Glyma07g05550* encoding a subunit of PDHC was found to be a DEG between high- and low-oil soybean accessions (Fig 2). A similar phenomenon between high- and low-oil accessions in oat was also observed by Hayden et al. [84]. Therefore, we assumed that the up-regulated expression of *PDHC* and *ACCase* in soybean likely resulted in the increase of carbon flux to FA synthesis, and then to an increase of the efficiency of FA synthesis [84].

### FA transportation, triacylglycerol synthesis and oil-body formation

#### FA transportation

Among the 54 dicot-specific genes involved in acyl-lipid metabolism, 12 genes encoded lipid transfer proteins (LTPs), including 5 DEGs between high- and low-oil soybean accessions (S2 Table). Apart from the above 12 high-oil dicot-specific LTPs, we also identified nine additional DEGs coding LTPs (S2 Table). Wang et al. [64] hypothesizes that an increase of the number of *LTP1* genes in sesame might enhance oil accumulation by strengthening the transport of FAs, acyl-CoAs, and other lipid molecules. On this basis, we proposed that abundant *LTP* genes in oil plants might possibly benefit oil accumulation. Among the 40 up-regulated genes during the stages from 5–6 mg to 400–500 mg (Fig 2), two genes *Glyma08g01180* and *Glyma06g11860* encoded ACP4 and LACS9, respectively. In addition, two genes (*Glyma13g11700* and *Glyma20g07280*) that belong to LACS family are up-regulated DEGs in high-oil accession (Fig 4B). Therefore, dicot-specific or up-regulation of *LTP*, *ACP4* and *LACS* in dicots could possibly increase the efficiency of plastidial fatty acid export for TAG synthesis and then consequently regulate seed oil content [55, 85, 86].

#### Triacylglycerol synthesis and oil-body formation

In TAG assembly, eight genes were highly up-regulated. Among these eight, *Glyma04g04060* encoding PP, *Glyma09g07520* and *Glyma13g16560 en*coding DGAT1, and *Glyma13g16790* encoding PDAT1 were also differentially expressed in high- and low-oil soybean accessions (Fig 2). *DGAT1* has a principal role in TAG biosynthesis [87] and over-expression of *DGAT1* had been shown to enhance oil accumulation [16, 32, 88]. In *B*. *napus*, two domestication-related genes *BnaA01g32210D* and *BnaAnng30990D*, encoding PP and DGAT1, respectively [89], possibly played roles in increasinging seed oil content. In oil-body formation, three and six genes respectively encoding STEROs and OLEs were identified and were up-regulated by some hundredfold between the stages from 5–6 mg to 400–500 mg (Fig 3 and S4 Dataset). Phylogenetic analysis showed more copies of genes coding OLEs in the dicots than in the grasses (Fig 3). Highly up-regulated and abundant OLEs possibly play vital roles in regulating seed oil content in dicots. Evidently, Siloto et al. [90] and Miquel et al. [51] showed that the size and spatial distribution of oil bodies affect the total lipid content and oil body proteins have specific functions in lipid accumulation.

We also found that 40 highly up-regulated genes and 28 DEGs in high- and low-oil soybean accessions were all enriched significantly in downstream section of the TAG biosynthesis pathway and the oil-body formation pathway (Figs 2 and 4B). This phenomenon was also observed in sesame [64]. Therefore, we deduced that enzymes in the downstream section of the TAG biosynthesis pathway and the oil-body formation pathway played vital roles in the variation of seed oil content in soybean and sesame [64]. In other words, the efficient flow of fatty acids to formation of TAG and oil-body may ultimately influence seed oil content.

### Signal transduction and other factors involved in lipid metabolism

Among the 54 dicot-specific genes involved in acyl-lipid metabolism (S2 Table), *Glyma07g01310*, *Glyma08g20710* and *Glyma15g02710* encode Phospholipase Dα (*PLDα*), which is able to hydrolyze phospholipids, producing signalling molecule phosphatidic acid [91]. A suppression of the expression of *PLDα* led to a significant decrease in triacylglycerol levels in *Arabidopsis* leaves [26] and could also slow the conversion of phosphatidylcholine to TAG in soybean seeds [92]. Two transgenic *B*. *napus* cultivars expressing an *Arabidopsis PLDα1* both demonstrated a 9% increase in seed total oil content [93]. In addition, *PLDα* is regulated by *GmMYB73*, which has been shown to be an important TF regulating lipid content [26]. Although these three dicot-specific *PLDα* are not DEGs in high- and low-oil soybean accessions, two of their homologous genes, *Glyma13g44170* and *Glyma08g22600*, are found to be differentially expressed genes (S2 Table). We suspect that up-regulated dicot-specific *PLDα* and the other two DEGs of *PLDα* would possibly have a significant effect on the seed oil content.

Among the 93 dicot-specific genes associated with acyl-lipid metabolism, 11 genes were in the category GO:0007165 (signal transduction) (Table 1 and S4 Table). Among these 11 genes, three (*Glyma09g07090*, *Glyma15g18380* and *Glyma17g06290*) encode GATA TFs, which play a role in light-mediated transcriptional regulation [94]. The three genes were homologous to the *Arabidopsis* gene *At5g56860* that is closely associated with lipid metabolism in green algae [95] and is capable of regulating carbon and nitrogen metabolism [96]. In this study, *Glyma15g18380* was validated to be a DEG in high- and low-oil soybean accessions. We deduced that these specific GATA TFs might affect the accumulation of seed oil in dicots. Two genes, *Glyma09g39570* and *Glyma10g12130*, are included in category PWY-5035 (gibberellin biosynthesis III) and might be related to the synthesis of the hormone gibberellin. Similarly, five dicot-specific genes, *Glyma04g02660*, *Glyma06g02690*, *Glyma14g40400*, *Glyma17g37750* and *Glyma17g37760* in GO:0005975 (carbohydrate metabolism) were annotated as PF02704 (Gibberellin regulated protein). The expression of genes encoding gibberellin regulated proteins is up-regulated by gibberellin [97]. It is known that the gibberellin signalling pathway is related to FA content [98]. More importantly, four genes (*Glyma04g02660*, *Glyma06g02690*, *Glyma17g37750* and *Glyma17g37760*) encoding gibberellin regulated proteins were also validated to be DEGs in high- and low-oil soybean accessions. Therefore, the above six gibberellin related genes may play roles in FA synthesis and thereby affect the oil content.

#### Other factors involved in lipid metabolism

Apart from the above specific genes directly participating in acyl-lipid metabolism, we also found that five dicot-specific genes in soybean were putatively involved in the lipid metabolic process (GO:0006629) (Table 1 and S4 Table). Among these genes, *Glyma02g45680* and *Glyma14g03130* encoded cytochrome P450, which plays a role in the FA metabolism in plants [99, 100]); and *Glyma18g45250* encoded a protein participating in glyceollin biosynthesis, which was related to lipid peroxidation [101]. Since these genes were highly expressed and coordinated with genes participating in acyl lipid synthesis, we hypothesized that these genes and their associated pathways possibly play some roles in seed oil accumulation.

### Conclusion

Ninety-three dicot-specific genes, including 42 and 27 genes respectively in carbohydrate degradation and transport, were predicted to be candidate genes associated with acyl-lipid metabolism pathways. And seed-specific TF genes *GmGRF5*, *ABI5* and *GmTZF4* were also predicted to play roles in regulating seed oil accumulation. Furthermore, *ACCase*, *DGAT1*, *PP*, *OLEs* and *STEROs* were highly up-regulated not only in specific stages of seed development but also in high-oil accessions, which indicates that enzymes in initial fatty acid synthesis, downstream of TAG biosynthesis and oil-body formation, played vital roles in the variation of seed oil content. In particular, highly up-regulated and abundant *OLEs* possibly play vital roles in determining seed oil content. Most of the above key genes were further confirmed by differential gene expression analysis between high-oil cultivated and low-oil wild soybeans.

## Materials and Methods

### Genomic data and gene expression data

The genomic data of four high-oil dicot species (*Glycine max* (seed oil content: 20%), *Gossypium raimondii* (30%), *Ricinus communis* (50%), and *Arabidopsis thaliana* (35%)) and three low-oil grasses (*Sorghum bicolor* (3%), *Setaria italic* (1.7%), and *Oryza sativa* (3%)) were downloaded from Phytozome V9.1 (http://www.phytozome.net/) [102]. The longest encoded protein sequence was chosen for genes with multiple transcripts.

The transcriptome data with two biological replicates of *G*. *max* Williams 82 [41] were downloaded from the Gene Expression Omnibus database (http://www.ncbi.nlm.nih.gov/geo/query/acc.cgi?acc=GSE42871). The data included seven stages of seed development: whole seed 4 DAF, whole seed 12–14 DAF, whole seed 22–24 DAF, whole seed 5–6 mg in weight, cotyledons 100–200 mg in weight, cotyledon 400–500 mg in weight, and dry whole seed.

### GO annotation and GO enrichment analysis

The GO annotations of the *G*. *max* genes, including molecular function, molecular location and biological process, were conducted using the online tool Goanna (http://agbase.msstate.edu/cgi-bin/tools/GOanna.cgi) [103]. The GO enrichment analysis was performed using GOstats with a threshold P value of less than 0.01 [104]. The GO slims, which are a subset of GO terms for a high level summary of the ontology content, were summarized using GOSlimViewer (http://www.agbase.msstate.edu/cgi-bin/tools/goslimviewer_select.pl) [103].

### OrthoMCL analysis and definition of lineage-specific clusters

Orthologous gene clusters were calculated from OrthoMCL comparisons of four dicots and three Grasses [40].

Based on all-against-all BLASTP comparisons of a set of protein sequences from genomes of interest, clusters of proteins were grouped according to reciprocal best similarity pairs between and within species, using OrthoMCL software implemented by the Markov Clustering algorithm (MCL; http://micans.org/mcl/) [105]. Here all the critical values were set as default values in the software. One OG was defined as dicot-specific if all the genes in the OG were coming from all the four rosid species but not from any grass species.

### Prediction of genes involved in lipid metabolism of *Glycine max*

More than 600 genes involved in acyl-lipid metabolism in Arabidopsis [7,8] were downloaded from the website ARALIP (http://aralip.plantbiology.msu.edu/). Based on the OrthoMCL result, all the orthologous and paralogous genes participating in acyl lipid biosynthesis in soybean were identified.

### Clustering by expression pattern and PLC network analysis

Using the transcriptome data, gene models were clustered using BioLayout Express^3D^, implemented by the MCL [42]; and any two genes with an absolute value of the Pearson correlation coefficient greater than 0.7 were considered to be similar in their expression level [43, 106].

The PLC network analysis developed by Wei et al. [43] was used to identify new candidate pathway members in the lipid synthesis pathway. Genes co-expressed with more than two genes in the soybean acyl-lipid metabolism pathway were considered as candidate lipid synthesis pathway members. A relatively stringent correlation threshold was set at the probability value of 1e-4.

### RNA-seq for transcriptome analysis of high- and low-oil soybean accessions

The materials used for RNA-seq to analyze lipid synthesis were three soybean accessions, one high-oil cultivar Handou 5 (HD5; seed oil content: 22.3%) and two low oil wild soybeans ZYD4364 (11.9%) and Y117249 (12.5%). Whole seeds at stages 15, 25, 35 and 55 DAF were harvested as samples. Total RNA of every sample was extracted from tissues using the TRIzol reagent (Invitrogen). The quality and the concentration of total RNA were quantified separately using Agilent 2200 TapeStation and ND-1000 Nanodrop. cDNA library were constructed using the same procedure described in Severin et al. [107] and sequenced using an Illumina HiSeq 2500 sequencing platform.

The raw reads were cleaned by removing reads with adapters and those of low quality. Clean reads were mapped to reference sequences using SOAPaligner/soap2 (http://soap.genomics.org.cn/soapdenovo.html). Mismatches no more than two bases were allowed in the alignment. The gene expression level was calculated by using RPKM method (Reads Per kb per Million reads) [108]. Fisher’s exact test method in DEGseq package [109] was used to identify DEGs between high- and low-oil accessions, at the 0.01 significant level.

## Supporting Information

S1 Dataset OGs of all the genes and genes in acyl-lipid metabolism of seven species. Dicot-specific OGs shared with all the four dicots and 4051 dicots-specific genes in soybean are also shown.
(XLSX)

S2 Dataset GO enrichment analysis in *Glycine max* for 4051 soybean genes in dicot-specific OGs. GOslim summary of the enriched GO terms are also listed.
(XLSX)

S3 Dataset GO enrichment analysis for 805 dicot-specific genes of clusters 3 to 5 in *Glycine max*. GOslim summary of the enriched GO terms are also listed.
(XLSX)

S4 Dataset Up-regulated genes of lipid synthesis core pathways in at least one of the seed developmental stages from 5–6 mg to 400–500 mg. (XLSX)

S5 Dataset Predicted dicot-specific genes and TFs that are associated with acyl-lipid metabolism and their co-expression network with acyl-lipid metabolism genes in soybean. Co-expression network of 19 known lipid-related transcription factors with core enzymes in lipid synthesis (P<0.05) are also shown.
(XLSX)

S1 Fig Co-expression network among soybean genes coding *ABI3*, *OLE* and *STERO*. Two genes with a coordinated relationship were linked by regular (P-value < 0.01) or bold (P-value < 1e-04) lines.
(PDF)

S2 Fig AW-boxes, with arrowhead, in 5’-upstream sequences of *GmWRI1* coordinated genes involved in lipid synthesis pathways. Schematic drawing of the sequence 2 kb upstream of the ATG start codon of *GmWRI1* co-expressed genes.
(PDF)

S3 Fig Phylogenetic analysis of *WRI1*. A: Phylogenetic tree of *WRI1* constructed by MEGA 6.0 using Neighbor-Joining method and the bootstrap test was performed with 1,000 iterations. Square boxes indicate duplication events and numbers on the branches represent the bootstrap support. Genes structure visualizing positions of exons and introns are also shown; this was constructed by GSDS 2.0. B: Selection detection using branch model implemented by PAML.
(PDF)

S1 Table Genomic information of the seven species used in this study. (PDF)

S2 Table Acyl-lipid metabolism genes that are high-oil dicot-specific or differentially expressed genes in high- and low-oil soybean accessions. (PDF)

S3 Table 207 genes in interested biological processes that were included in the 805 genes in clusters 3 to 5. (PDF)

S4 Table Annotation and differential expression analysis of 93 dicot-specific genes that were associated with oil accumulation. (PDF)

S5 Table 327 lipid-related TFs predicted by PLC analysis. (PDF)
