# Supplementary material for: An Integrated Bioinformatics Analysis Reveals Divergent Evolutionary Pattern of Oil Biosynthesis in High- and Low-Oil Plants
Source: PLoS One. 2016 May 9;11(5):e0154882. doi: 10.1371/journal.pone.0154882 (PMC4861283; doi:10.1371/journal.pone.0154882)
Supplement: S1 Fig — Two genes with a coordinated relationship were linked by regular (P-value < 0.01) or bold (P-value < 1e-04) lines. (PDF) [file pone.0154882.s006.pdf]

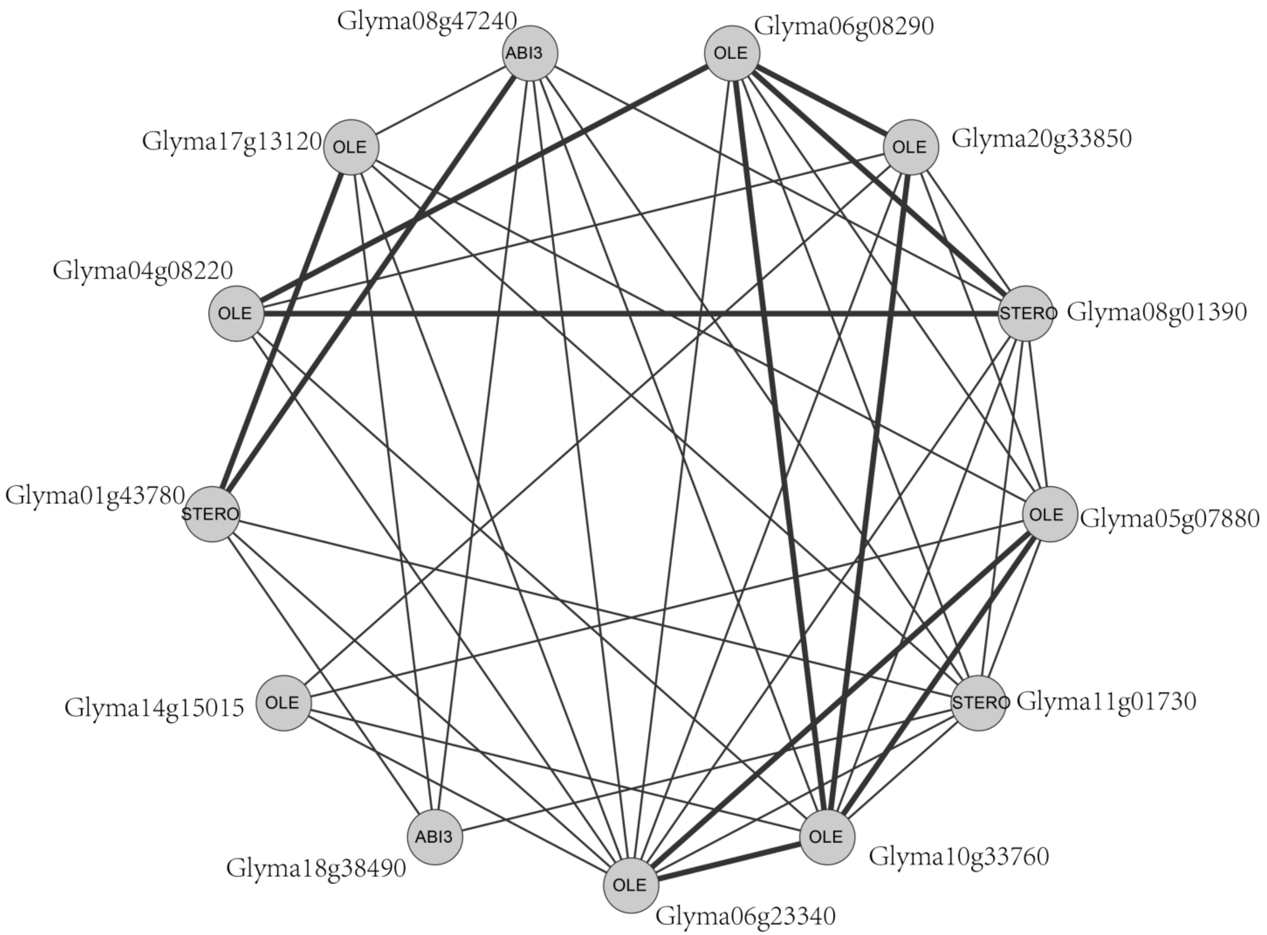

S1 Fig. Co-expression network among soybean genes coding *ABI3*, *OLE* and *STERO*.

Two genes with a coordinated relationship were linked by regular (P-value < 0.01) or bold lines (P-value < 1E-04).
