# Supplementary material for: An Integrated Bioinformatics Analysis Reveals Divergent Evolutionary Pattern of Oil Biosynthesis in High- and Low-Oil Plants
Source: PLoS One. 2016 May 9;11(5):e0154882. doi: 10.1371/journal.pone.0154882 (PMC4861283; doi:10.1371/journal.pone.0154882)
Supplement: S2 Fig — Schematic drawing of the sequence 2 kb upstream of the ATG start codon of GmWRI1 co-expressed genes. (PDF) [file pone.0154882.s007.pdf]

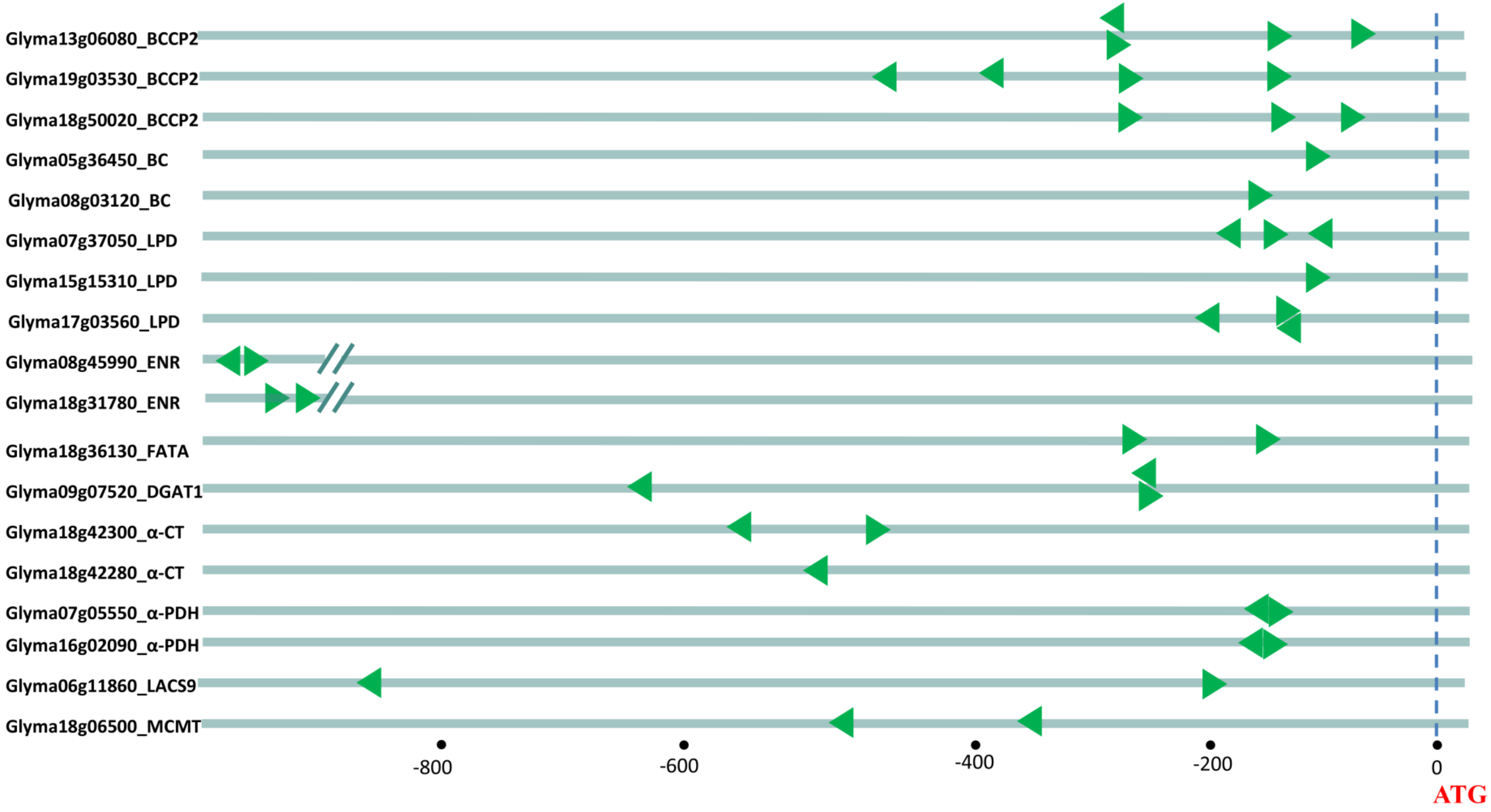

S2 Fig. AW-boxes, with arrowhead, in 5'-upstream sequences of *GmWRI1* coordinated genes involved in lipid synthesis pathways.

Schematic drawing of the sequence 2 kb upstream of the ATG start codon of *GmWRI1* co-expressed genes.
