# Supplementary material for: An Integrated Bioinformatics Analysis Reveals Divergent Evolutionary Pattern of Oil Biosynthesis in High- and Low-Oil Plants
Source: PLoS One. 2016 May 9;11(5):e0154882. doi: 10.1371/journal.pone.0154882 (PMC4861283; doi:10.1371/journal.pone.0154882)
Supplement: S3 Fig — A: Phylogenetic tree of WRI1 constructed by MEGA 6.0 using Neighbor-Joining method and the bootstrap test was performed with 1,000 iterations. Square boxes indicate duplication events and numbers on the branches represent the bootstrap support. Genes structure visualizing positions of exons and introns are also shown; this was constructed by GSDS 2.0. B: Selection detection using branch model implemented by PAML. (PDF) [file pone.0154882.s008.pdf]

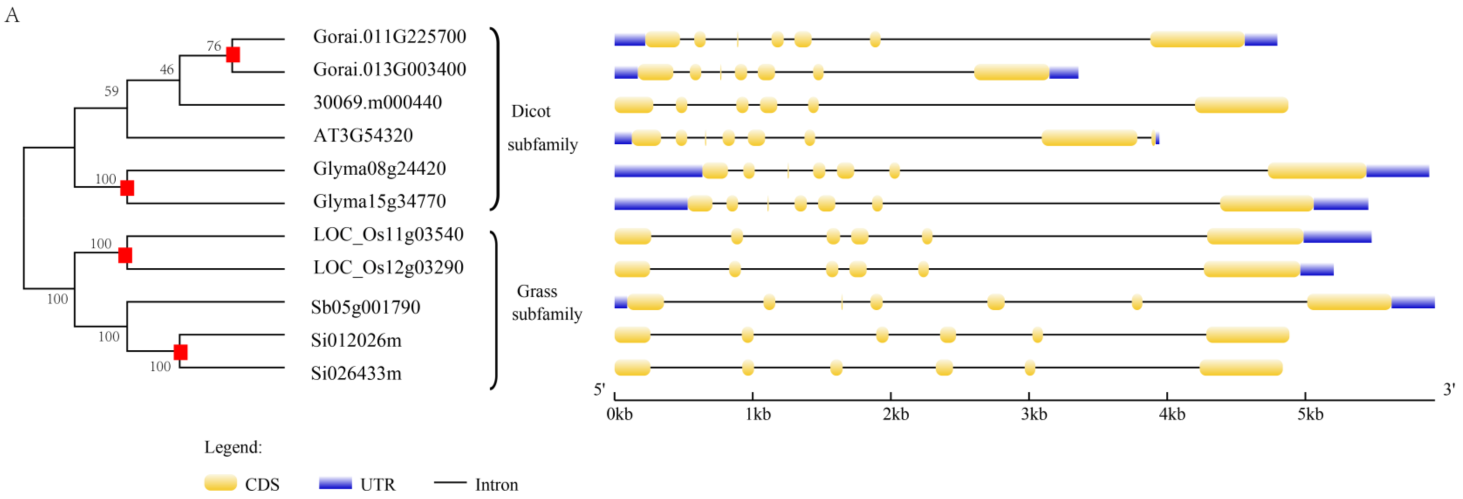

B

Parameters and summary statistics for detecting selection of *WRI1* using branch-model by PAML

Model	ω setting	-ln L	Estimated parameters	LRT
One-ratio	Entire tree:ω_0	-10323.522	ω_0=0.2623	
Two-ratio	dicot subfamily: ω1<br>grass subfamily&orther branches: ω0	-10317.045	ω0=0.2809<br>ω1=0.0861	P=3.192E-04** (two ratio vs. one ratio)

S3 Fig. Phylogenetic analysis of *WRI1*

A: Phylogenetic tree of *WRI1* constructed by MEGA 6.0 using Neighbor-Joining method and the bootstrap test was performed with 1,000 iterations. Square boxes indicated duplication events and numbers on the branches represented the bootstrap support. Genes structure visualizing positions of exons and introns were also showed which was constructed by GSDS 2.0. B: Selection detection using branch model implemented by PAML.
