# Supplementary material for: An Integrated Bioinformatics Analysis Reveals Divergent Evolutionary Pattern of Oil Biosynthesis in High- and Low-Oil Plants
Source: PLoS One. 2016 May 9;11(5):e0154882. doi: 10.1371/journal.pone.0154882 (PMC4861283; doi:10.1371/journal.pone.0154882)
Supplement: S1 Table — (PDF) [file pone.0154882.s009.pdf]

S1 Table. Genomic information of the seven species used in this study

**Common name**	**Species name**	**Release version**	**No. of genes**	**Access**
Soybean	*Glycine max*	V 1.1	54,175	http://genome.jgi.doe.gov/pages/dynamicOrganismDownload.jsf?organism=PhytozomeV9
Arabidopsis	*Arabidopsis thaliana*	TAIR 10	27,416	ftp://ftp.arabidopsis.org/home/tair/Genes/
cotton	*Gossypium raimondii*	V 2.1	37,505	http://genome.jgi.doe.gov/pages/dynamicOrganismDownload.jsf?organism=PhytozomeV9
Castor bean	*Ricinus communis*	V 0.1	31,221	ftp://ftp.tigr.org/pub/data/castorbean/release_0.1/
Foxail millet	*Setaria italica*	V 2.1	35,471	http://genome.jgi.doe.gov/pages/dynamicOrganismDownload.jsf?organism=PhytozomeV9
Sorghum	*Sorghum bicolor*	V 1.0	27,608	http://genome.jgi.doe.gov/pages/dynamicOrganismDownload.jsf?organism=PhytozomeV9
Rice	*Oryza sativa*	V 7.0	39,047	http://rapdb.dna.affrc.go.jp/download/irgsp1.html/
