# Supplementary material for: An Integrated Bioinformatics Analysis Reveals Divergent Evolutionary Pattern of Oil Biosynthesis in High- and Low-Oil Plants
Source: PLoS One. 2016 May 9;11(5):e0154882. doi: 10.1371/journal.pone.0154882 (PMC4861283; doi:10.1371/journal.pone.0154882)
Supplement: S2 Table — (PDF) [file pone.0154882.s010.pdf]

S2 Table. Acyl-lipid metabolism genes that are high-oil dicot-specific or differently expressed genes in high- and low-oil soybean accessions

Pathway	Specific gene	Arabidopsis homolog	Enzyme/Protein	Abbreviation	Dicot-specific cluster	P-value in differential expression analysis		
						Group H-L1	Group H-L2	Significance at the 0.01 level
Cutin Synthesis & Transport 1	*Glyma03g01070*	*AT4G00400*	Glycerol-3-Phosphate Acyltransferase	GPAT8	Cluster 1	1.30E-02	5.19E-01	
Cutin Synthesis & Transport 1	*Glyma07g07580*	*AT4G00400*	Glycerol-3-Phosphate Acyltransferase	GPAT8	Cluster 1	5.95E-02	2.80E-01	
Cutin Synthesis & Transport 1	*Glyma04g07140*	*AT5G25390*	SHN Transcription Factors	SHN3	Cluster 1	5.08E-01	NA	
Cutin Synthesis & Transport 1	*Glyma06g07240*	*AT5G11190*	SHN Transcription Factors	SHN2	Cluster 1	1.00E+00	1.00E+00	
Cutin Synthesis & Transport 1	*Glyma15g01140*	*AT1G15360*	SHN Transcription Factors	WIN1/SHN1	Cluster 1	NA	NA	
Cutin Synthesis & Transport 1	*Glyma17g31900*	*AT5G25390*	SHN Transcription Factors	SHN3	Cluster 2	1.00E+00	2.57E-01	
Cutin Synthesis & Transport 1	*Glyma08g01890*	*AT1G63710*	Fatty Acyl omega-Hydroxylase (CYP86A)	CYP86A7		5.29E-16	1.49E-07	TRUE
Cutin Synthesis & Transport 1	*Glyma18g54010*	*AT2G33510*	A WW domain-containing protein	CFL1		7.02E-06	3.42E-06	TRUE
Eukaryotic Galactolipid & Sulfolipid Synthesis	*Glyma04g04060*	*AT5G42870*	Phosphatidate Phosphatase	PP		1.07E-07	4.89E-06	TRUE
Eukaryotic Galactolipid & Sulfolipid Synthesis	*Glyma04g37360*	*AT3G48610*	Phospholipase C (Non specific)	NPC6 (Non specific)		2.32E-04	4.20E-03	TRUE
Eukaryotic Galactolipid & Sulfolipid Synthesis	*Glyma20g08030*	*AT2G26870*	Phospholipase C (Non specific)	NPC2 (Non specific)		3.78E-08	1.16E-03	TRUE
Eukaryotic Phospholipid Synthesis & Editing	*Glyma02g08600*	*AT1G74320*	Choline Kinase	CK		5.41E-07	1.02E-03	TRUE
Eukaryotic Phospholipid Synthesis & Editing	*Glyma03g30070*	*AT3G12120*	Oleate Desaturase	FAD2		1.08E-13	8.38E-14	TRUE
Eukaryotic Phospholipid Synthesis & Editing	*Glyma04g04060*	*AT5G42870*	Phosphatidate Phosphatase	PP		1.07E-07	4.89E-06	TRUE
Eukaryotic Phospholipid Synthesis & Editing	*Glyma18g02210*	*AT2G22240*	myo-inositol-3-phosphate synthase	MIPS2		6.84E-08	9.29E-18	TRUE
Fatty Acid Elongation & Wax Biosynthesis	*Glyma04g07140*	*AT5G25390*	SHN Transcription Factors	SHN3	Cluster 1	5.08E-01	NA	
Fatty Acid Elongation & Wax Biosynthesis	*Glyma06g07240*	*AT5G11190*	SHN Transcription Factors	SHN2	Cluster 1	1.00E+00	1.00E+00	
Fatty Acid Elongation & Wax Biosynthesis	*Glyma15g01140*	*AT1G15360*	SHN Transcription Factors	WIN1/SHN1	Cluster 1	NA	NA	
Fatty Acid Elongation & Wax Biosynthesis	*Glyma17g31900*	*AT5G25390*	SHN Transcription Factors	SHN3	Cluster 2	1.00E+00	2.57E-01	

Fatty Acid Elongation & Wax Biosynthesis	*Glyma10g43800*	*AT5G04530*	Ketoacyl-CoA Synthase	KCS19	Cluster 1	5.08E-01	1.00E+00	
Fatty Acid Elongation & Wax Biosynthesis	*Glyma08g13750*	*AT1G24470*	Ketoacyl-CoA Reductase	KCR2	Cluster 3	NA	NA	
Fatty Acid Elongation & Wax Biosynthesis	*Glyma18g41590*	*AT5G55410*	Lipid Transfer Protein	LTP	Cluster 1	6.06E-08	1.00E+00	
Fatty Acid Elongation & Wax Biosynthesis	*Glyma20g10310*	*AT3G43720*	Lipid Transfer Protein	LTP	Cluster 1	5.08E-01	5.04E-01	
Fatty Acid Elongation & Wax Biosynthesis	*Glyma07g17000*	*AT5G55410*	Lipid Transfer Protein	LTP	Cluster 1	NA	NA	
Fatty Acid Elongation & Wax Biosynthesis	*Glyma07g17030*	*AT5G55410*	Lipid Transfer Protein	LTP	Cluster 1	1.14E-30	8.96E-21	TRUE
Fatty Acid Elongation & Wax Biosynthesis	*Glyma08g36180*	*AT2G37870*	Lipid Transfer Protein	(LTP type 5)	Cluster 3	5.83E-04	8.06E-04	TRUE
Fatty Acid Elongation & Wax Biosynthesis	*Glyma16g18700*	*AT2G37870*	Lipid Transfer Protein	(LTP type 5)	Cluster 3	1.69E-03	3.65E-04	TRUE
Fatty Acid Elongation & Wax Biosynthesis	*Glyma18g09550*	*AT5G55460*	Lipid Transfer Protein	LTP	Cluster 4	NA	NA	
Fatty Acid Elongation & Wax Biosynthesis	*Glyma14g03660*	*AT4G33355*	Lipid Transfer Protein	LTP	Cluster 4	4.02E-11	3.20E-23	TRUE
Fatty Acid Elongation & Wax Biosynthesis	*Glyma09g06100*	*AT4G33355*	Lipid Transfer Protein	LTP	Cluster 4	5.34E-01	1.41E-01	
Fatty Acid Elongation & Wax Biosynthesis	*Glyma09g06130*	*AT4G33355*	Lipid Transfer Protein	LTP	Cluster 4	5.75E-08	4.71E-05	TRUE
Fatty Acid Elongation & Wax Biosynthesis	*Glyma13g03810*	*AT3G43720*	Lipid Transfer Protein	LTP	Cluster 4	4.69E-01	3.95E-01	
Fatty Acid Elongation & Wax Biosynthesis	*Glyma16g26480*	*AT5G13900*	Lipid Transfer Protein	LTP	Cluster 4	NA	NA	
Fatty Acid Elongation & Wax Biosynthesis	*Glyma03g04920*	*AT2G38540*	Lipid Transfer Protein type 1	(LTP type 1)		1.18E-189	4.05E-155	TRUE
Fatty Acid Elongation & Wax Biosynthesis	*Glyma03g16620*	*AT3G53980*	Lipid Transfer Protein type 4	(LTP type 4)		1.85E-04	4.12E-04	TRUE
Fatty Acid Elongation & Wax Biosynthesis	*Glyma05g09160*	*AT3G18280*	Lipid Transfer Protein type 2	(LTP type 2)		5.25E-123	6.44E-173	TRUE
Fatty Acid Elongation & Wax Biosynthesis	*Glyma10g44500*	*AT5G48490*	Lipid Transfer Protein type 3	(LTP type 3)		9.74E-03	6.17E-29	TRUE
Fatty Acid Elongation & Wax Biosynthesis	*Glyma11g36460*	*AT1G55260*	Lipid Transfer Protein type 5	(LTP type 5)		4.38E-05	1.38E-09	TRUE
Fatty Acid Elongation & Wax Biosynthesis	*Glyma17g14620*	*AT1G62790*	Lipid Transfer Protein type 5	(LTP type 5)		8.05E-03	3.63E-03	TRUE
Fatty Acid Elongation & Wax Biosynthesis	*Glyma18g53150*	*AT3G18280*	Lipid Transfer Protein type 2	(LTP type 2)		1.46E-04	1.78E-06	TRUE
Fatty Acid Elongation & Wax Biosynthesis	*Glyma19g00710*	*AT3G18280*	Lipid Transfer Protein type 2	(LTP type 2)		2.13E-206	0.00E+00	TRUE
Fatty Acid Elongation & Wax Biosynthesis	*Glyma20g39271*	*AT5G48485*	Lipid Transfer Protein type 3	(LTP type 3)		8.02E-98	2.14E-77	TRUE
Fatty Acid Elongation & Wax Biosynthesis	*Glyma03g38810*	*AT1G09430*	ATP Citrate Lyase A subunit	ACLA-3		2.71E-05	9.16E-07	TRUE

Fatty Acid Elongation & Wax Biosynthesis	*Glyma06g11120*	*AT1G36160*	Acetyl-CoA Carboxylase	ACC1		9.87E-06	3.54E-03	TRUE
Fatty Acid Elongation & Wax Biosynthesis	*Glyma10g41110*	*AT2G01320*	ABC Transporter	WBC7 / ABCG7		2.66E-07	8.91E-03	TRUE
Fatty Acid Elongation & Wax Biosynthesis	*Glyma20g28010*	*AT4G04890*	Homeodomain glabrous1	HDG1		8.51E-06	9.16E-07	TRUE
Fatty Acid Elongation, Desaturation & Export From Plastid	*Glyma02g15600*	*AT2G43710*	Stearoyl-ACP Desaturase	FAB2		5.57E-37	1.52E-33	TRUE
Fatty Acid Elongation, Desaturation & Export From Plastid	*Glyma07g32850*	*AT2G43710*	Stearoyl-ACP Desaturase	FAB2		8.96E-12	1.37E-04	TRUE
Fatty Acid Elongation, Desaturation & Export From Plastid	*Glyma14g27990*	*AT1G43800*	Stearoyl-ACP Desaturase	DES6		0.00E+00	0.00E+00	TRUE
Fatty Acid Elongation, Desaturation & Export From Plastid	*Glyma13g11700*	*AT2G04350*	Long-Chain Acyl-CoA Synthetase	LACS8		7.08E-04	2.22E-04	TRUE
Fatty Acid Elongation, Desaturation & Export From Plastid	*Glyma20g07280*	*AT2G04350*	Long-Chain Acyl-CoA Synthetase	LACS8		8.45E-04	2.80E-03	TRUE
Fatty Acid Elongation, Desaturation & Export From Plastid	*Glyma17g05200*	*AT1G74960*	Ketoacyl-ACP Synthase II	KASII		3.59E-05	1.82E-03	TRUE
Fatty Acid Synthesis	*Glyma09g38440*	*AT5G16390*	Biotin Carboxyl Carrier Protein; subunit of Heteromeric ACCase	BCCP1	Cluster 1	4.30E-01	6.34E-01	
Fatty Acid Synthesis	*Glyma13g06080*	*AT5G15530*	Biotin Carboxyl Carrier Protein; subunit of Heteromeric ACCase	BCCP2	Cluster 3	3.70E-01	7.71E-02	
Fatty Acid Synthesis	*Glyma18g50020*	*AT5G15530*	Biotin Carboxyl Carrier Protein; subunit of Heteromeric ACCase	BCCP2	Cluster 3	3.80E-02	2.74E-01	
Fatty Acid Synthesis	*Glyma19g03530*	*AT5G15530*	Biotin Carboxyl Carrier Protein; subunit of Heteromeric ACCase	BCCP2	Cluster 3	7.50E-29	5.92E-37	TRUE
Fatty Acid Synthesis	*Glyma05g36450*	*AT5G35360*	Biotin Carboxylase; subunit of Heteromeric ACCase	BC	Cluster 3	5.52E-02	6.08E-01	
Fatty Acid Synthesis	*Glyma08g03120*	*AT5G35360*	Biotin Carboxylase; subunit of Heteromeric ACCase	BC	Cluster 3	1.60E-01	3.41E-01	
Fatty Acid Synthesis	*Glyma08g24420*	*AT3G54320*	AP2/EREBP Transcription Factors	WRI1	Cluster 3	8.19E-02	1.00E+00	
Fatty Acid Synthesis	*Glyma15g34770*	*AT3G54320*	AP2/EREBP Transcription Factors	WRI1	Cluster 3	4.33E-02	9.44E-02	
Fatty Acid Synthesis	*Glyma18g42280*	*AT2G38040*	Carboxyltransferase alpha Subunit of Heteromeric ACCase	alpha-CT	Cluster 3	5.99E-02	6.68E-01	
Fatty Acid Synthesis	*Glyma18g42300*	*AT2G38040*	Carboxyltransferase alpha Subunit of Heteromeric ACCase	alpha-CT	Cluster 3	8.33E-01	4.24E-01	
Fatty Acid Synthesis	*Glyma07g05550*	*AT1G01090*	Pyruvate Dehydrogenase alpha subunit, E1a component of Pyruvate Dehydrogenase Complex	PDH (E1 alpha)		5.10E-05	7.51E-03	TRUE
Fatty Acid Synthesis	*Glyma15g05800*	*AT5G10160*	Hydroxyacyl-ACP Dehydrase	HAD		1.77E-03	2.82E-04	TRUE

Fatty Acid Synthesis; Prokaryotic Galactolipid, Sulfolipid, & Phospholipid Synthesis 1	*Glyma02g15600*	*AT2G43710*	Stearoyl-ACP Desaturase	FAB2		5.57E-37	1.52E-33	TRUE
Fatty Acid Synthesis; Prokaryotic Galactolipid, Sulfolipid, & Phospholipid Synthesis 1	*Glyma07g32850*	*AT2G43710*	Stearoyl-ACP Desaturase	FAB2		8.96E-12	1.37E-04	TRUE
Fatty Acid Synthesis; Prokaryotic Galactolipid, Sulfolipid, & Phospholipid Synthesis 1	*Glyma14g27990*	*AT1G43800*	Stearoyl-ACP Desaturase	DES6		0.00E+00	0.00E+00	TRUE
Fatty Acid Synthesis; Prokaryotic Galactolipid, Sulfolipid, & Phospholipid Synthesis 1	*Glyma20g07280*	*AT2G04350*	Long-Chain Acyl-CoA Synthetase	LACS8		8.45E-04	2.80E-03	TRUE
Fatty Acid Synthesis; Prokaryotic Galactolipid, Sulfolipid, & Phospholipid Synthesis 1	*Glyma13g11700*	*AT2G04350*	Long-Chain Acyl-CoA Synthetase	LACS8		7.08E-04	2.22E-04	TRUE
Fatty Acid Synthesis; Prokaryotic Galactolipid, Sulfolipid, & Phospholipid Synthesis 1	*Glyma17g05200*	*AT1G74960*	Ketoacyl-ACP Synthase II	KASII		3.59E-05	1.82E-03	TRUE
Lipid Trafficking	*Glyma09g41040*	*AT5G04930*	Aminophospholipid ATPase	ALA1		1.15E-07	1.43E-04	TRUE
Mitochondrial Lipopolysaccharide Synthesis	*Glyma08g10600*	*AT1G30370*	sn-1-specific acylhydrolase	DLAH	Cluster 1	1.00E+00	1.00E+00	
Oxylipin Metabolism 1	*Glyma02g16600*	*AT3G63200*	Acyl-Hydrolase (Patatin-like)	PAH	Cluster 1	7.44E-02	8.90E-01	
Oxylipin Metabolism 1	*Glyma10g03230*	*AT3G63200*	Acyl-Hydrolase (Patatin-like)	PAH	Cluster 1	4.11E-01	1.23E-03	
Oxylipin Metabolism 1	*Glyma11g06070*	*AT4G37070*	Acyl-Hydrolase (Patatin-like)	PAH	Cluster 1	1.00E+00	NA	
Oxylipin Metabolism 1	*Glyma14g07890*	*AT1G19640*	Jasmonic Acid Carboxyl Methyltransferase	JCMT	Cluster 4	NA	NA	
Oxylipin Metabolism 1	*Glyma01g44600*	*AT1G76690*	Oxo-Phytodienoic Acid Reductase	OPR		3.00E-06	1.32E-14	TRUE
Oxylipin Metabolism 1; Oxylipin Metabolism 2	*Glyma08g10600*	*AT1G30370*	Acylhydrolase (DAD1-like)	AH	Cluster 1	1.00E+00	1.00E+00	
Oxylipin Metabolism 1; Oxylipin Metabolism 2	*Glyma03g41770*	*AT3G61680*	Lipid Acylhydrolase-like	LAH		1.13E-04	3.07E-07	TRUE
Oxylipin Metabolism 1; Oxylipin Metabolism 2	*Glyma07g03910*	*AT1G55020*	Lipoxygenase	LOX		5.43E-27	4.61E-13	TRUE
Oxylipin Metabolism 1; Oxylipin Metabolism 2	*Glyma13g42310*	*AT1G55020*	Lipoxygenase	LOX		0.00E+00	0.00E+00	TRUE
Oxylipin Metabolism 1; Oxylipin Metabolism 2	*Glyma13g42320*	*AT1G55020*	Lipoxygenase	LOX		0.00E+00	0.00E+00	TRUE
Oxylipin Metabolism 1; Oxylipin Metabolism 2	*Glyma15g03030*	*AT1G55020*	Lipoxygenase	LOX		0.00E+00	0.00E+00	TRUE
Oxylipin Metabolism 1; Oxylipin Metabolism 2	*Glyma15g03040*	*AT1G55020*	Lipoxygenase	LOX		2.08E-08	1.33E-08	TRUE

Pathway, function or subcellular location uncertain	*Glyma09g36030*	*AT3G51970*	Membrane-bound O-acyltransferase	ACAT	Cluster 1	3.25E-01	1.00E+00	
Pathway, function or subcellular location uncertain	*Glyma08g20330*	*AT1G17500*	Translocase	TL		1.75E-06	3.13E-06	TRUE
Pathway, function or subcellular location uncertain	*Glyma09g01080*	*AT4G04020*	Plastid Lipid-associated Protein	LP		2.07E-20	1.30E-46	TRUE
Pathway, function or subcellular location uncertain	*Glyma15g11910*	*AT4G04020*	Plastid Lipid-associated Protein	LP		1.56E-36	1.34E-177	TRUE
Pathway, function or subcellular location uncertain	*Glyma17g08680*	*AT1G74210*	Glycerophosphoryl Diester Phosphodiesterase	GPDEPDE		2.08E-08	8.56E-04	TRUE
Phospholipid Signaling	*Glyma08g26330*	*AT5G39400*	Phosphoinositide 3-Phosphatase	PI3P	Cluster 1	5.85E-01	1.00E+00	
Phospholipid Signaling	*Glyma08g22420*	*AT1G52700*	Lysophospholipase	LPLA	Cluster 1	7.07E-01	4.20E-01	
Phospholipid Signaling	*Glyma13g43990*	*AT1G52700*	Lysophospholipase	LPLA	Cluster 1	7.14E-03	2.61E-02	
Phospholipid Signaling	*Glyma15g01350*	*AT1G52700*	Lysophospholipase	LPLA	Cluster 1	8.70E-02	5.60E-01	
Phospholipid Signaling	*Glyma07g03670*	*AT1G52700*	Lysophospholipase	LPLA	Cluster 2	1.00E+00	1.26E-01	
Phospholipid Signaling	*Glyma07g01310*	*AT1G55180*	Phospholipase D alpha	PLD alpha	Cluster 3	4.37E-01	1.00E+00	
Phospholipid Signaling	*Glyma08g20710*	*AT1G55180*	Phospholipase D alpha	PLD alpha	Cluster 6	NA	1.00E+00	
Phospholipid Signaling	*Glyma15g02710*	*AT1G55180*	Phospholipase D alpha	PLD alpha	Cluster 6	NA	1.00E+00	
Phospholipid Signaling	*Glyma13g44170*	*AT3G15730*	Phospholipase D alpha	PLD alpha		1.12E-04	3.45E-03	TRUE
Phospholipid Signaling	*Glyma08g22600*	*AT3G15730*	Phospholipase D alpha	PLD alpha		1.13E-06	5.83E-04	TRUE
Phospholipid Signaling	*Glyma11g08640*	*AT4G35790*	Phospholipase D delta	PLD delta		8.89E-05	2.68E-03	TRUE
Phospholipid Signaling	*Glyma03g27610*	*AT2G40850*	Phosphatidylinositol-4-Kinase gamma	PI4K gamma	Cluster 6	NA	NA	
Phospholipid Signaling	*Glyma02g13700*	*AT2G03890*	Phosphatidylinositol-4-Kinase gamma	PI4K gamma		2.50E-12	3.08E-05	TRUE
Phospholipid Signaling	*Glyma18g51100*	*AT2G03890*	Phosphatidylinositol-4-Kinase gamma	PI4K gamma		1.28E-16	5.93E-05	TRUE
Phospholipid Signaling	*Glyma05g33310*	*AT5G07360*	Fatty Acid Amide Hydrolase	FAAH		1.56E-13	2.38E-11	TRUE
Phospholipid Signaling	*Glyma13g17510*	*AT3G14270*	Phosphatidylinositol-Phosphate Kinase type III	PIPK-III		1.22E-05	2.35E-03	TRUE

Phospholipid Signaling	*Glyma15g05150*	*AT1G60890*	Phosphatidylinositol-Phosphate Kinase type IB	PIPK-IB		9.71E-04	4.64E-03	TRUE
Prokaryotic Galactolipid, Sulfolipid, & Phospholipid Synthesis 2	*Glyma07g03370*	*AT3G15850*	FAD5-like Desaturase	FAD5 like (ADS)	Cluster 2	1.00E+00	2.31E-01	
Prokaryotic Galactolipid, Sulfolipid, & Phospholipid Synthesis 2	*Glyma08g22730*	*AT3G15850*	FAD5-like Desaturase	FAD5 like (ADS)	Cluster 3	2.64E-01	1.56E-01	
Sphingolipid Biosynthesis 1	*Glyma02g11820*	*AT2G46210*	Sphingobase-D8 Desaturase	SLD		3.61E-14	1.74E-14	TRUE
Sphingolipid Biosynthesis 2	*Glyma18g52070*	*AT1G27980*	Dihydrosphingosine Phosphate Lyase	DPL1		3.37E-18	4.96E-11	TRUE
Suberin Synthesis & Transport 1	*Glyma04g07140*	*AT5G25390*	SHN Transcription Factors	SHN3	Cluster 1	5.08E-01	NA	
Suberin Synthesis & Transport 1	*Glyma17g31900*	*AT5G25390*	SHN Transcription Factors	SHN3	Cluster 2	1.00E+00	2.57E-01	
Suberin Synthesis & Transport 1	*Glyma01g05890*	*AT1G14190*	omega-Hydroxy Fatty Acyl Dehydrogenase	HFADH	Cluster 2	1.00E+00	1.00E+00	
Suberin Synthesis & Transport 1	*Glyma02g12060*	*AT1G14190*	omega-Hydroxy Fatty Acyl Dehydrogenase	HFADH	Cluster 2	1.00E+00	1.00E+00	
Suberin Synthesis & Transport 1	*Glyma05g03580*	*AT1G12570*	omega-Hydroxy Fatty Acyl Dehydrogenase	HFADH		1.81E-07	5.36E-03	TRUE
Suberin Synthesis & Transport 2	*Glyma08g10930*	*AT1G67990*	Caffeoyl-CoA O-Methyltransferase	CCoAOMT	Cluster 3	8.49E-04	1.19E-02	
Suberin Synthesis & Transport 3	*Glyma16g26480*	*AT5G13900*	Lipid Transfer Protein	LTP	Cluster 4	NA	NA	
Triacylglycerol & Fatty Acid Degradation	*Glyma18g52590*	*AT1G65520*	Enoyl CoA isomerase	ECI1	Cluster 1	6.68E-01	8.90E-01	
Triacylglycerol & Fatty Acid Degradation	*Glyma08g06110*	*AT1G73480*	Monoacylglycerol Lipase (MAGL)	MAGL		4.28E-10	1.49E-17	TRUE
Triacylglycerol & Fatty Acid Degradation	*Glyma09g32430*	*AT4G29010*	Multifunctional Protein	AIM1		2.09E-13	4.78E-08	TRUE
Triacylglycerol & Fatty Acid Degradation	*Glyma13g31670*	*AT4G14440*	Enoyl CoA isomerase	ECI3		9.90E-03	1.34E-05	TRUE
Triacylglycerol Biosynthesis	*Glyma08g24420*	*AT3G54320*	AP2/EREBP Transcription Factors (or is it AP2/ERWEBP)	WRI1	Cluster 3	8.19E-02	1.00E+00	
Triacylglycerol Biosynthesis	*Glyma15g34770*	*AT3G54320*	AP2/EREBP Transcription Factors (or is it AP2/ERWEBP)	WRI1	Cluster 3	4.33E-02	9.44E-02	
Triacylglycerol Biosynthesis	*Glyma16g05480*	*AT3G26790*	Transcriptional factor with high similarity to the B3 region of the VP1/ABI3-like proteins	FUS3	Cluster 3	4.21E-03	1.72E-02	
Triacylglycerol Biosynthesis	*Glyma11g31234*	*AT2G30470*	a member of a novel family of B3 domain proteins	HSI2/VAL1		2.14E-06	2.37E-03	TRUE
Triacylglycerol Biosynthesis	*Glyma02g08600*	*AT1G74320*	Choline Kinase	CK		5.41E-07	1.02E-03	TRUE
Triacylglycerol Biosynthesis	*Glyma03g30070*	*AT3G12120*	Oleate Desaturase	FAD2		1.08E-13	8.38E-14	TRUE
Triacylglycerol Biosynthesis	*Glyma04g04060*	*AT5G42870*	Phosphatidate Phosphatase	PP		1.07E-07	4.89E-06	TRUE

Triacylglycerol Biosynthesis	*Glyma01g43780*	*AT4G10020*	Steroleosin	STERO		7.16E-34	5.19E-63	TRUE
Triacylglycerol Biosynthesis	*Glyma11g01730*	*AT4G10020*	Steroleosin	STERO		1.66E-57	1.29E-68	TRUE
Triacylglycerol Biosynthesis	*Glyma08g01390*	*AT5G50600*	Steroleosin	STERO		1.60E-13	1.46E-08	TRUE
Triacylglycerol Biosynthesis	*Glyma08g47240*	*AT3G24650*	Homologous to the maize transcription factor Viviparous-1	ABI3		1.22E-65	8.85E-30	TRUE
Triacylglycerol Biosynthesis	*Glyma09g07520*	*AT2G19450*	Acyl-CoA : Diacylglycerol Acyltransferase	DGAT1		1.92E-07	8.60E-04	TRUE
Triacylglycerol Biosynthesis	*Glyma13g16560*	*AT2G19450*	Acyl-CoA : Diacylglycerol Acyltransferase	DGAT1		3.43E-10	3.46E-07	TRUE
Triacylglycerol Biosynthesis	*Glyma13g16790*	*AT5G13640*	Phospholipid : Diacylglycerol Acyltransferase	PDAT1		5.38E-03	4.93E-04	TRUE
Triacylglycerol Biosynthesis	*Glyma17g13120*	*AT3G18570*	Oil-Body Oleosin	OBO		1.95E-55	3.83E-37	TRUE
Triacylglycerol Biosynthesis	*Glyma20g33850*	*AT4G25140*	Oil-Body Oleosin	OBO		1.26E-08	1.94E-34	TRUE
Triacylglycerol Biosynthesis	*Glyma10g33760*	*AT4G25140*	Oil-Body Oleosin	OBO		0.00E+00	0.00E+00	TRUE
Triacylglycerol Biosynthesis	*Glyma04g08220*	*AT2G25890*	Oil-Body Oleosin	OBO		0.00E+00	0.00E+00	TRUE
Triacylglycerol Biosynthesis	*Glyma05g07880*	*AT3G18570*	Oil-Body Oleosin	OBO		2.61E-60	1.73E-75	TRUE
Triacylglycerol Biosynthesis	*Glyma06g08290*	*AT2G25890*	Oil-Body Oleosin	OBO		0.00E+00	0.00E+00	TRUE
Triacylglycerol Biosynthesis	*Glyma05g08880*	*AT3G01570*	Oil-Body Oleosin	OBO/ole4	Cluster 2	7.38E-09	1.83E-43	TRUE
Triacylglycerol Biosynthesis	*Glyma19g00400*	*AT3G01570*	Oil-Body Oleosin	OBO/ole4	Cluster 2	4.40E-184	9.32E-70	TRUE

Among the 1123 genes in acyl-lipid metabolism, 55 were found to be high-oil dicot-specific and their corresponding clusters were also listed, and 77 were differentially expressed in high and low-oil soybean accessions.

Group H-L1: high-oil HD5 and low-oil ZYD4364; Group H-L2: HD5 and low-oil Y117249. Genes significantly and differently expressed in the two groups were indicated by “TRUE”.
