# Supplementary material for: An Integrated Bioinformatics Analysis Reveals Divergent Evolutionary Pattern of Oil Biosynthesis in High- and Low-Oil Plants
Source: PLoS One. 2016 May 9;11(5):e0154882. doi: 10.1371/journal.pone.0154882 (PMC4861283; doi:10.1371/journal.pone.0154882)
Supplement: S3 Table — (PDF) [file pone.0154882.s011.pdf]

S3 Table. 207 genes in interested biological processes among the 805 genes in clusters 3 to 5

GO slim	GO ID	Term	P-value	Gene model
GO:0005975 Carbohydrate metabolic process	GO:0032885	regulation of polysaccharide biosynthetic process	2.66E-03	*Glyma0165s00200, Glyma01g28520, Glyma01g33440, Glyma01g33480, Glyma01g33500, Glyma01g37150, Glyma01g43490, Glyma02g34870, Glyma02g36150, Glyma02g43230, Glyma02g47500, Glyma03g03400, Glyma03g03460, Glyma03g08820, Glyma03g08850, Glyma03g08860, Glyma03g08910, Glyma03g08930, Glyma03g08980, Glyma03g36250, Glyma04g02660, Glyma04g03990, Glyma04g11230, Glyma04g37650, Glyma04g40630, Glyma05g02600, Glyma05g06410, Glyma05g36680, Glyma06g02690, Glyma06g04170, Glyma06g09120, Glyma06g09860, Glyma06g10970, Glyma06g11700, Glyma06g14160, Glyma06g47690, Glyma07g05970, Glyma07g11420, Glyma07g30860, Glyma08g06440, Glyma08g16880, Glyma09g01520, Glyma09g04330, Glyma09g30820, Glyma09g36660, Glyma10g08740, Glyma10g10530, Glyma10g16100, Glyma11g08120, Glyma11g19310, Glyma11g36640, Glyma12g00700, Glyma12g28890, Glyma12g30480, Glyma12g31180, Glyma12g36630, Glyma13g02860, Glyma13g27310, Glyma13g32090, Glyma13g39130, Glyma14g01260, Glyma14g06280, Glyma14g08820, Glyma14g08970, Glyma14g22970, Glyma14g34640, Glyma14g35620, Glyma14g40110, Glyma14g40400, Glyma15g08960, Glyma15g12490, Glyma15g15360, Glyma16g00330, Glyma17g09260, Glyma17g36200, Glyma17g37750, Glyma17g37760, Glyma18g00560, Glyma19g05080, Glyma19g07830, Glyma19g25570, Glyma19g35380, Glyma19g38900, Glyma19g42510, Glyma19g44720*
	GO:0044264	cellular polysaccharide metabolic process	1.71E-04	
	GO:0034637	cellular carbohydrate biosynthetic process	4.76E-05	
	GO:1900030	regulation of pectin biosynthetic process	1.84E-05	
	GO:0045492	xylan biosynthetic process	6.61E-08	
	GO:0043255	regulation of carbohydrate biosynthetic process	2.26E-03	
	GO:0046835	carbohydrate phosphorylation	2.30E-07	
	GO:0045489	pectin biosynthetic process	2.37E-02	
	GO:0005983	starch catabolic process	1.29E-05	
	GO:0033692	cellular polysaccharide biosynthetic process	1.93E-05	
	GO:0045491	xylan metabolic process	9.57E-08	
	GO:0033530	raffinose metabolic process	1.01E-02	
	GO:0010413	glucuronoxylan metabolic process	6.61E-08	
	GO:0005976	polysaccharide metabolic process	4.45E-05	
	GO:0032881	regulation of polysaccharide metabolic process	1.75E-02	
	GO:0044262	cellular carbohydrate metabolic process	4.41E-04	
	GO:0042353	fucose biosynthetic process	1.93E-02	
	GO:0070592	cell wall polysaccharide biosynthetic process	4.21E-08	
	GO:0010383	cell wall polysaccharide metabolic process	1.74E-08	

	GO:0044275	cellular carbohydrate catabolic process	4.22E-04	
	GO:0052541	plant-type cell wall cellulose metabolic process	2.73E-02	
	GO:0006109	regulation of carbohydrate metabolic process	2.84E-04	
	GO:0009251	glucan catabolic process	1.29E-05	
	GO:0080091	regulation of raffinose metabolic process	2.22E-03	
	GO:0006004	fucose metabolic process	2.87E-02	
	GO:0010410	hemicellulose metabolic process	5.46E-08	
	GO:0016051	carbohydrate biosynthetic process	4.53E-03	
	GO:0000271	polysaccharide biosynthetic process	3.53E-06	
	GO:0044247	cellular polysaccharide catabolic process	1.29E-05	
GO:0006629 Lipid metabolic process	GO:0046890	regulation of lipid biosynthetic process	1.25E-03	*Glyma02g45680, Glyma02g46220, Glyma04g04050, Glyma06g04220, Glyma08g26670, Glyma09g40580, Glyma14g02510, Glyma14g03130, Glyma18g45250, Glyma18g45260*
	GO:0090032	negative regulation of steroid hormone biosynthetic process	1.64E-03	
	GO:0010423	negative regulation of brassinosteroid biosynthetic process	1.64E-03	
	GO:0050810	regulation of steroid biosynthetic process	1.63E-05	
	GO:0042616	paclitaxel metabolic process	6.97E-04	
	GO:0010894	negative regulation of steroid biosynthetic process	1.64E-03	
	GO:0019216	regulation of lipid metabolic process	3.87E-02	
	GO:0019218	regulation of steroid metabolic process	1.63E-05	
	GO:0090030	regulation of steroid hormone biosynthetic process	1.63E-05	
	GO:0045939	negative regulation of steroid metabolic process	1.64E-03	
	GO:0010422	regulation of brassinosteroid biosynthetic process	1.63E-05	
GO:0006810	GO:0035436	triose phosphate transmembrane transport	2.66E-03	*Glyma01g20790, Glyma01g31520, Glyma01g37750, Glyma03g05880,*

Transport	GO:0015714	phosphoenolpyruvate transport	1.48E-03	*Glyma03g22060, Glyma03g22120, Glyma03g32040, Glyma03g33150, Glyma03g33660, Glyma03g35430, Glyma04g06230, Glyma04g40100, Glyma05g02600, Glyma05g25540, Glyma05g31200, Glyma05g36700, Glyma05g37050, Glyma05g37060, Glyma05g37440, Glyma06g06190, Glyma06g06270, Glyma06g13520, Glyma06g14750, Glyma06g22240, Glyma06g26370, Glyma06g36840, Glyma06g37040, Glyma06g37050, Glyma06g40690, Glyma06g40710, Glyma06g40780, Glyma06g40950, Glyma06g40980, Glyma06g41430, Glyma07g12460, Glyma07g38800, Glyma07g38830, Glyma08g02510, Glyma08g02840, Glyma08g08520, Glyma08g08530, Glyma08g14390, Glyma08g16380, Glyma08g36190, Glyma08g44050, Glyma08g45410, Glyma09g03130, Glyma09g03290, Glyma09g04870, Glyma09g06260, Glyma09g06330, Glyma09g08410, Glyma09g33570, Glyma11g07560, Glyma12g04500, Glyma12g07510, Glyma12g13070, Glyma12g23290, Glyma12g33880, Glyma12g34020, Glyma13g27680, Glyma13g31530, Glyma14g23930, Glyma14g33680, Glyma15g01720, Glyma15g11270, Glyma15g13630, Glyma15g14210, Glyma15g15990, Glyma15g17310, Glyma16g01050, Glyma16g03780, Glyma17g01920, Glyma17g09260, Glyma17g09640, Glyma18g08740, Glyma18g14810, Glyma19g34820, Glyma19g35870, Glyma19g36370, Glyma19g44720, Glyma20g23120, Glyma20g39140*
	GO:0051223	regulation of protein transport	1.90E-02	
	GO:0006612	protein targeting to membrane	3.54E-04	
	GO:0042306	regulation of protein import into nucleus	3.64E-04	
	GO:0032940	secretion by cell	4.54E-02	
	GO:0015688	iron chelate transport	5.56E-03	
	GO:0006863	purine nucleobase transport	6.19E-03	
	GO:0015851	nucleobase transport	6.19E-03	
	GO:0015760	glucose-6-phosphate transport	5.13E-04	
	GO:0042873	aldonate transport	2.22E-03	
	GO:0046822	regulation of nucleocytoplasmic transport	5.67E-03	
	GO:0015713	phosphoglycerate transport	2.22E-03	
	GO:1901678	iron coordination entity transport	5.56E-03	
	GO:0015712	hexose phosphate transport	5.13E-04	
	GO:0032386	regulation of intracellular transport	2.73E-02	
	GO:0015717	triose phosphate transport	2.66E-03	
	GO:0033157	regulation of intracellular protein transport	1.48E-02	
GO:0007165 Signal transduction	GO:0009740	gibberellic acid mediated signaling pathway	3.01E-02	*Glyma02g47500, Glyma04g40100, Glyma06g09120, Glyma06g14750, Glyma07g11300, Glyma08g41200, Glyma09g07090, Glyma09g30930, Glyma09g39570, Glyma10g04890, Glyma10g12130, Glyma11g35270, Glyma12g32910, Glyma13g19250, Glyma13g29070, Glyma13g37550, Glyma13g39340, Glyma14g01260, Glyma15g09980, Glyma15g18380, Glyma17g06290, Glyma18g03130*
	GO:2000022	regulation of jasmonic acid mediated signaling pathway	1.65E-02	
	GO:0010017	red or far-red light signaling pathway	3.62E-02	
	GO:0010476	gibberellin mediated signaling pathway	3.01E-02	
GO:0009056	GO:0042436	indole-containing compound catabolic process	2.07E-02	*Glyma0165s00200, Glyma01g28520, Glyma01g31630, Glyma03g08820,*

Catabolic process	GO:0009074	aromatic amino acid family catabolic process	2.11E-02	*Glyma03g08850, Glyma03g08860, Glyma03g08910, Glyma03g08930, Glyma03g08980, Glyma04g02660, Glyma05g29170, Glyma06g02690, Glyma08g26670, Glyma12g33880, Glyma13g36630, Glyma14g40400, Glyma15g08960, Glyma17g37750, Glyma17g37760, Glyma18g43830*
	GO:0005983	starch catabolic process	1.29E-05	
	GO:0006569	tryptophan catabolic process	1.81E-02	
	GO:0044275	cellular carbohydrate catabolic process	4.22E-04	
	GO:0046218	indolalkylamine catabolic process	1.81E-02	
	GO:0009251	glucan catabolic process	1.29E-05	
	GO:0044247	cellular polysaccharide catabolic process	1.29E-05	
GO:0019538 Protein metabolic process	GO:0006417	regulation of translation	2.92E-03	*Glyma01g03580, Glyma05g23620, Glyma06g10110, Glyma07g19540, Glyma08g39290, Glyma10g42660, Glyma13g22620, Glyma17g12200, Glyma17g16690, Glyma18g19720, Glyma20g00850*
