# Supplementary material for: An Integrated Bioinformatics Analysis Reveals Divergent Evolutionary Pattern of Oil Biosynthesis in High- and Low-Oil Plants
Source: PLoS One. 2016 May 9;11(5):e0154882. doi: 10.1371/journal.pone.0154882 (PMC4861283; doi:10.1371/journal.pone.0154882)
Supplement: S4 Table — (PDF) [file pone.0154882.s012.pdf]

S4 Table. Annotation and differential expression analysis of 93 dicots-specific genes that were associated with oil accumulation

GO slims	Pathways	Enzymes	Genes	Pfam ID	Arabidopsis homologs	Definition	P-value in differential expression analysis		
							Group H-L1	Group H-L2	Significance at the 0.01 level
GO:0005975 carbohydrate metabolic process			*Glyma0165s00200*	PF00686	*AT5G01260*	Carbohydrate-binding-like fold	NA	NA	
			Glyma01g28520	PF00686	*AT5G01260*	Carbohydrate-binding-like fold	2.40E-02	8.44E-03	
			Glyma03g08860	PF00686	*AT5G01260*	Carbohydrate-binding-like fold	NA	NA	
	PWY-6902; chitin degradation II	chitinase	*Glyma09g04330*	PF00182	*AT3G16920*	chitinase-like protein 2	1.25E-05	8.18E-04	TRUE
	PWY-6902; chitin degradation II	chitinase	*Glyma15g15360*	PF00182	*AT3G16920*	chitinase-like protein 2	8.50E-02	8.51E-01	
			Glyma18g00560	PF03016	*AT4G13990*	Exostosin family protein	NA	5.04E-01	
			Glyma02g47500	PF00646	*AT5G48170*	F-box family protein	2.50E-01	4.25E-02	
			Glyma14g01260	PF00646	*AT5G48170*	F-box family protein	1.81E-03	1.28E-04	TRUE
			Glyma17g09260	PF08022, PF01794, PF08030	*AT5G50160*	ferric reduction oxidase 8	6.59E-01	1.00E+00	
			Glyma04g02660	PF02704	*AT1G75750*	GAST1 protein homolog 1	3.59E-03	2.94E-11	TRUE
			Glyma06g02690	PF02704	*AT1G75750*	GAST1 protein homolog 1	3.74E-05	8.83E-06	TRUE
			Glyma14g40400	PF02704	*AT1G75750*	GAST1 protein homolog 1	NA	NA	
			Glyma17g37760	PF02704	*AT1G75750*	GAST1 protein homolog 1	6.43E-05	2.15E-06	TRUE

			Glyma17g37750	PF02704	*AT2G18420*	Gibberellin-regulated family protein	7.11E-09	2.50E-11	TRUE
			Glyma02g43230	PF02458	*AT2G40230*	HXXXD-type acyl-transferase family protein	1.39E-01	1.00E+00	
			Glyma14g22970	PF00847	*AT1G21910*	Integrase-type DNA-binding superfamily protein	6.59E-01	1.00E+00	
			Glyma10g16100	PF00612	*AT5G03960*	IQ-domain 12	4.37E-01	1.00E+00	
			Glyma07g05970	PF00394, PF07731, PF07732	*AT2G46570*	laccase 6	4.14E-01	1.00E+00	
			Glyma04g40630	PF03168	*AT5G53730*	Late embryogenesis abundant (LEA) hydroxyproline-rich glycoprotein family	4.03E-03	1.89E-02	
			Glyma06g14160	PF03168	*AT5G53730*	Late embryogenesis abundant (LEA) hydroxyproline-rich glycoprotein family	4.14E-01	1.00E+00	
			Glyma05g06410	PF00249	*AT5G14340*	myb domain protein 40	1.00E+00	1.00E+00	
			Glyma19g07830	PF00249	*AT5G14340*	myb domain protein 40	1.00E+00	NA	
	PWY-1081; homogalacturonan degradation	pectinesterase	*Glyma01g33440*	PF01095, PF04043	*AT2G45220*	Plant invertase/pectin methylesterase inhibitor superfamily	NA	NA	
	PWY-1081; homogalacturonan degradation	pectinesterase	*Glyma03g03400*	PF01095, PF04043	*AT2G45220*	Plant invertase/pectin methylesterase inhibitor superfamily	NA	NA	
	PWY-1081; homogalacturonan degradation	pectinesterase	*Glyma03g03460*	PF04043, PF01095	*AT2G45220*	Plant invertase/pectin methylesterase inhibitor superfamily	NA	NA	
	PWY-1081; homogalacturonan degradation	pectinesterase	*Glyma06g47690*	PF04043, PF01095	*AT2G45220*	Plant invertase/pectin methylesterase inhibitor superfamily	NA	NA	
	PWY-1081; homogalacturonan degradation	pectinesterase	*Glyma12g00700*	PF04043, PF01095	*AT3G47400*	Plant invertase/pectin methylesterase inhibitor superfamily	1.00E+00	1.00E+00	

			Glyma01g37150		*AT2G17940*	Plant protein of unknown function (DUF827)	2.29E-01	7.92E-01	
			Glyma11g08120		*AT2G17940*	Plant protein of unknown function (DUF827)	8.59E-04	6.29E-03	TRUE
			Glyma15g08960	PF05097	*AT1G29240*	Protein of unknown function (DUF688)	8.70E-02	3.64E-01	
			Glyma05g36680	PF00097	*AT4G24015*	RING/U-box superfamily protein	2.29E-01	3.98E-01	
			Glyma14g40110	PF00097	*AT5G42200*	RING/U-box superfamily protein	3.49E-04	1.60E-02	
			Glyma02g34870	PF06886	*AT2G35880*	TPX2 (targeting protein for Xklp2) protein family	2.73E-05	6.10E-05	TRUE
			Glyma03g36250	PF06886	*AT2G35880*	TPX2 (targeting protein for Xklp2) protein family	1.00E+00	1.00E+00	
			Glyma10g10530	PF06886	*AT2G35880*	TPX2 (targeting protein for Xklp2) protein family	1.85E-09	1.57E-09	TRUE
			Glyma19g38900	PF06886	*AT2G35880*	TPX2 (targeting protein for Xklp2) protein family	4.15E-06	4.11E-03	TRUE
			Glyma02g36150	PF04535	*AT2G36100*	Uncharacterised protein family (UPF0497)	NA	NA	
			Glyma10g08740	PF04535	*AT2G36100*	Uncharacterised protein family (UPF0497)	NA	NA	
			Glyma08g16880				6.88E-01	1.88E-01	
			Glyma09g01520		*AT3G09950*		1.91E-01	2.05E-01	
			Glyma17g36200		*AT1G47410*		1.46E-01	6.09E-01	
			Glyma14g08970		*AT1G47410*		7.38E-03	5.64E-02	
GO:0006629 lipid metabolic			*Glyma02g45680*	PF00067	*AT2G42850*	cytochrome P450, family 718	1.75E-01	4.20E-01	
			Glyma14g03130	PF00067	*AT2G42850*	cytochrome P450, family 718	NA	NA	

process	PWY-2463; medicarpin biosynthesis PWY-2464;maackiain biosynthesis; PWY-2761; glyceollin biosynthesis I	pterocarpin synthase	*Glyma18g45250*	PF01370	*AT2G45400*	NAD(P)-binding Rossmann-fold superfamily protein	1.37E-01	2.57E-01	
			Glyma02g46220	PF04885	*AT1G50650*	Stigma-specific Stig1 family protein	1.81E-23	1.19E-41	TRUE
			Glyma14g02510	PF04885	*AT1G50650*	Stigma-specific Stig1 family protein	1.00E+00	2.32E-09	
GO:0006810 transport	PWY-2161; folate polyglutamylation	tetrahydrofolate synthase	*Glyma12g13070*	PF00069	*AT4G04960*	Concanavalin A-like lectin protein kinase family protein	NA	NA	
			Glyma08g02510	PF07765	*AT5G41790*	COP1-interactive protein 1	1.16E-04	1.69E-03	TRUE
			Glyma03g05880	PF01582, PF00560, PF00931	*AT5G46260*	disease resistance protein (TIR-NBS-LRR class) family	NA	NA	
			Glyma15g17310	PF00931, PF01582, PF07725	*AT5G41750*	Disease resistance protein (TIR-NBS-LRR class) family	5.85E-01	5.93E-01	
			Glyma06g40980	PF00931, PF01582, PF07725 ,PF00560	*AT5G17680*	disease resistance protein (TIR-NBS-LRR class), putative	4.78E-02	2.02E-01	
			Glyma07g12460	PF01582, PF00931, PF07725	*AT5G17680*	disease resistance protein (TIR-NBS-LRR class), putative	1.00E+00	4.53E-01	
			Glyma09g06260	PF01582, PF00931	*AT5G17680*	disease resistance protein (TIR-NBS-LRR class), putative	NA	NA	
			Glyma12g34020	PF01582, PF00560, PF00931	*AT5G17680*	disease resistance protein (TIR-NBS-LRR class), putative	4.37E-01	1.00E+00	
			Glyma18g14810	PF01582, PF07725, PF00931	*AT5G17680*	disease resistance protein (TIR-NBS-LRR class), putative	1.00E+00	4.53E-01	
			Glyma09g04870	PF03242	*AT4G15910*	drought-induced 21	1.00E+00	7.40E-01	
			Glyma15g15990	PF03242	*AT4G15910*	drought-induced 21	4.38E-05	3.10E-41	TRUE

			Glyma18g08740	PF03151	*AT4G18220*	Drug/metabolite transporter superfamily protein	3.25E-01	1.00E+00	
			Glyma20g23120	PF00646	*AT2G39490*	F-box family protein	5.50E-01	1.52E-01	
	PWY-5934; iron reduction and absorption	ferric-chelate reductase (NADH)	*Glyma17g09260*	PF08022, PF01794, PF08030	*AT5G50160*	ferric reduction oxidase 8	6.59E-01	1.00E+00	
			Glyma07g38830	PF03151, PF00892	*AT1G61800*	glucose 6-phosphate/phosphate translocator 2	NA	NA	
			Glyma13g27680	PF03151, PF00892	*AT1G61800*	glucose-6-phosphate/phosphate translocator 2	2.29E-01	1.00E+00	
			Glyma15g11270	PF00892, PF03151	*AT1G61800*	glucose-6-phosphate/phosphate translocator 2	1.27E-22	6.42E-24	TRUE
			Glyma04g06230		*AT2G24762*	glutamine dumper 4	5.08E-01	NA	
			Glyma03g33150	PF00403	*AT5G02600*	Heavy metal transport/detoxification superfamily protein	2.29E-02	1.89E-02	
			Glyma17g09640	PF00249	*AT4G18770*	myb domain protein 98	1.00E+00	NA	
			Glyma05g37050	PF07765	*AT1G64330*	myosin heavy chain-related	9.58E-05	4.20E-03	TRUE
			Glyma09g08410	PF04043	*AT5G20740*	Plant invertase/pectin methylesterase inhibitor superfamily protein	4.29E-03	1.65E-02	
			Glyma12g33880	PF05097	*AT2G30990*	Protein of unknown function (DUF688)	4.84E-02	1.52E-01	
			Glyma19g34820	PF00514, PF04564	*AT2G23140*	RING/U-box superfamily protein with ARM repeat domain	1.26E-02	7.78E-02	
			Glyma03g33660	PF03094	*AT3G45290*	Seven transmembrane MLO family protein	NA	1.00E+00	
			Glyma06g22240	PF03634	*AT5G60970*	TEOSINTE BRANCHED 1, cycloidea and PCF transcription factor 5	4.14E-01	1.00E+00	

			Glyma14g33680		*AT1G36940*		4.37E-01	4.53E-01	
GO:0007165 Signal transduction	PWY-5035; gibberellin biosynthesis III (early C-13 hydroxylation)	gibberellin 3β-dioxygenase	*Glyma09g39570*	PF03171	*AT4G23340*	2-oxoglutarate (2OG) and Fe(II)-dependent oxygenase superfamily protein	NA	NA	
			Glyma10g12130	PF03171	*AT1G52790*	2-oxoglutarate (2OG) and Fe(II)-dependent oxygenase superfamily protein	5.08E-01	NA	
	PWY-6220; jasmonoyl-amino acid conjugates biosynthesis I<br>PWY-6233; jasmonoyl-amino acid conjugates biosynthesis II	jasmonoyl-leucine synthetase/ jasmonoyl-valine synthetase/ jasmonyl-isoleucine synthetase	*Glyma12g32910*	PF03321	*AT4G03400*	Auxin-responsive GH3 family protein	1.00E+00	5.04E-01	
			Glyma13g39340	PF00170	*AT2G36270*	Basic-leucine zipper (bZIP) transcription factor family protein	6.78E-09	1.21E-05	TRUE
			Glyma02g47500	PF00646	*AT5G48170*	F-box family protein	2.50E-01	4.25E-02	
			Glyma14g01260	PF00646	*AT5G48170*	F-box family protein	1.81E-03	1.28E-04	TRUE
			Glyma09g07090	PF00320	*AT5G56860*	GATA type zinc finger transcription factor family protein	5.85E-01	1.25E-01	
			Glyma15g18380	PF00320	*AT5G56860*	GATA type zinc finger transcription factor family protein	2.87E-07	3.85E-07	TRUE
			Glyma17g06290	PF00320	*AT5G56860*	GATA type zinc finger transcription factor family protein	2.91E-01	2.67E-02	
			Glyma13g29070	PF09425, PF06200	*AT1G30135*	jasmonate-zim-domain protein 8	1.00E+00	1.00E+00	
			Glyma15g09980	PF09425, PF06200	*AT1G30135*	jasmonate-zim-domain protein 8	5.85E-01	1.00E+00	
GO:0009056 Catabolic process			*Glyma0165s00200*	PF00686	*AT5G01260*	Carbohydrate-binding-like fold	NA	NA	
			Glyma01g28520	PF00686	*AT5G01260*	Carbohydrate-binding-like fold	2.40E-02	8.44E-03	

			Glyma03g08860	PF00686	*AT5G01260*	Carbohydrate-binding-like fold	NA	NA	
			Glyma04g02660	PF02704	*AT1G75750*	GAST1 protein homolog 1	3.59E-03	2.94E-11	TRUE
			Glyma06g02690	PF02704	*AT1G75750*	GAST1 protein homolog 1	3.74E-05	8.83E-06	TRUE
			Glyma14g40400	PF02704	*AT1G75750*	GAST1 protein homolog 1	NA	NA	
			Glyma17g37760	PF02704	*AT1G75750.1*	GAST1 protein homolog 1	6.43E-05	2.15E-06	TRUE
			Glyma17g37750	PF02704	*AT2G18420*	Gibberellin-regulated family protein	7.11E-09	2.50E-11	TRUE
			Glyma05g29170	PF05097	*AT1G29240*	Protein of unknown function (DUF688)	1.00E+00	1.00E+00	
			Glyma12g33880	PF05097	*AT2G30990*	Protein of unknown function (DUF688)	4.84E-02	1.52E-01	
			Glyma15g08960	PF05097	*AT1G29240*	Protein of unknown function (DUF688)	8.70E-02	3.64E-01	
GO:0019538 Protein metabolic process			*Glyma05g23620*		*AT4G17810*	C2H2 and C2HC zinc fingers superfamily protein	3.52E-04	2.62E-02	
			Glyma17g16690		*AT4G17810*	C2H2 and C2HC zinc fingers superfamily protein	2.50E-01	2.37E-01	
			Glyma01g03580	PF04690	*AT1G23420*	Plant-specific transcription factor YABBY family protein	NA	1.00E+00	
			Glyma06g10110	PF04690	*AT1G23420*	Plant-specific transcription factor YABBY family protein	NA	NA	
			Glyma08g39290	PF04690	*AT1G23420*	Plant-specific transcription factor YABBY family protein	NA	NA	

			Glyma17g12200	PF04690	*AT2G26580*	plant-specific transcription factor YABBY family protein	1.96E-02	9.10E-01	
			Glyma18g19720	PF04690	*AT1G23420*	Plant-specific transcription factor YABBY family protein	NA	1.00E+00	

Group H-L1: high-oil HD5 and low-oil ZYD4364; Group H-L2: HD5 and low-oil Y117249. Genes significantly and differently expressed in the two groups were indicated by "TRUE".
