# Supplementary material for: An Integrated Bioinformatics Analysis Reveals Divergent Evolutionary Pattern of Oil Biosynthesis in High- and Low-Oil Plants
Source: PLoS One. 2016 May 9;11(5):e0154882. doi: 10.1371/journal.pone.0154882 (PMC4861283; doi:10.1371/journal.pone.0154882)
Supplement: S5 Table — (PDF) [file pone.0154882.s013.pdf]

S5 Table. 327 lipid-related TFs predicted by PLC analysis

Subfamily	TF	Dicot-specific	P-value in differential expression analysis				Subfamily	TF	Dicot-specific	P-value in differential expression analysis		
			DEHD5 – ZYD4364	DEHD5 – Y117249	Significance at the 0.01 level					DEHD5 – ZYD4364	DEHD5 – Y117249	Significance at the 0.01 level
AP2	*Glyma08g24420*	TRUE	8.19E-02	1.00E+00			G2-like	*Glyma19g32850*		5.85E-01	5.93E-01	
AP2	*Glyma15g34770*	TRUE	4.33E-02	9.44E-02			G2-like	*Glyma20g24290*		1.00E+00	7.64E-01	
AP2	*Glyma03g29240*		1.79E-02	9.65E-02			GATA	*Glyma13g00200*	TRUE	7.03E-02	7.23E-01	
AP2	*Glyma03g33470*		7.74E-01	7.79E-01			GATA	*Glyma14g36150*	TRUE	3.06E-02	7.72E-02	
AP2	*Glyma12g07800*		1.00E+00	4.72E-01			GATA	*Glyma17g34670*		5.38E-03	8.42E-04	TRUE
AP2	*Glyma13g00950*		6.18E-01	1.31E-26			GATA	*Glyma03g39220*		2.56E-02	7.31E-01	
AP2	*Glyma17g07010*		1.41E-01	1.65E-16			GATA	*Glyma06g10290*		8.27E-01	6.90E-01	
ARF	*Glyma04g43350*	TRUE	1.66E-03	8.80E-01			GATA	*Glyma07g30140*		2.62E-01	8.65E-01	
ARF	*Glyma20g32040*		3.09E-01	5.95E-01			GRAS	*Glyma18g04500*		2.93E-59	6.66E-27	TRUE
ARR-B	*Glyma07g08590*		6.82E-02	1.56E-01			GRAS	*Glyma04g21340*		2.21E-01	1.00E+00	
ARR-B	*Glyma17g33230*		6.59E-01	6.64E-01			GRAS	*Glyma04g43090*		2.41E-01	6.32E-01	
B3	*Glyma20g01130*	TRUE	6.73E-02	6.56E-01			GRAS	*Glyma05g03020*		NA	5.04E-01	
B3	*Glyma16g05110*		1.00E+00	1.00E+00			GRAS	*Glyma11g14730*		NA	NA	
B3	*Glyma18g05840*		3.56E-01	3.50E-01			GRAS	*Glyma13g02840*		2.29E-01	5.57E-01	
B3	*Glyma18g38490*		9.51E-15	1.26E-01			GRAS	*Glyma14g01960*		2.05E-01	1.02E-02	
BES1	*Glyma06g03700*		1.35E-03	2.05E-03	TRUE		GRAS	*Glyma15g15110*		4.37E-01	1.00E+00	
BES1	*Glyma04g03610*		1.76E-02	1.90E-01			GRAS	*Glyma17g13680*		NA	1.00E+00	
BES1	*Glyma18g46630*		3.26E-01	1.00E+00			GRAS	*Glyma17g17400*		2.72E-01	5.36E-01	
bHLH	*Glyma04g34080*	TRUE	NA	1.32E-01			GRF	*Glyma07g04290*		2.20E-13	3.15E-08	TRUE
bHLH	*Glyma05g01590*		5.98E-05	5.11E-03	TRUE		GRF	*Glyma19g37740*	TRUE	1.00E+00	3.65E-01	

bHLH	*Glyma06g20000*		1.69E-04	3.04E-12	TRUE		GRF	*Glyma14g10090*		1.95E-02	3.34E-01	
bHLH	*Glyma06g20400*	TRUE	NA	NA			HD-ZIP	*Glyma20g28010*		8.51E-06	9.16E-07	TRUE
bHLH	*Glyma08g26110*	TRUE	1.02E-02	1.95E-04			HD-ZIP	*Glyma02g02290*		9.71E-04	1.87E-01	
bHLH	*Glyma09g33590*		1.57E-06	4.58E-05	TRUE		HD-ZIP	*Glyma04g40960*		5.29E-01	2.66E-02	
bHLH	*Glyma10g01010*	TRUE	5.14E-01	3.92E-01			HD-ZIP	*Glyma05g04990*		5.79E-01	4.36E-03	
bHLH	*Glyma11g05920*	TRUE	2.63E-04	1.45E-11	TRUE		HD-ZIP	*Glyma05g30000*		7.62E-02	1.15E-01	
bHLH	*Glyma11g33840*	TRUE	NA	NA			HD-ZIP	*Glyma06g13890*		1.00E+00	3.65E-01	
bHLH	*Glyma13g41670*	TRUE	5.35E-01	1.41E-03			HD-ZIP	*Glyma06g46000*		5.03E-01	5.50E-01	
bHLH	*Glyma17g19830*	TRUE	3.95E-01	5.01E-03			HD-ZIP	*Glyma07g08340*		7.44E-02	8.86E-01	
bHLH	*Glyma18g04420*	TRUE	1.00E+00	5.04E-01			HD-ZIP	*Glyma08g13110*		1.46E-01	8.04E-01	
bHLH	*Glyma20g22010*	TRUE	8.12E-01	1.59E-01			HD-ZIP	*Glyma08g21890*		4.79E-01	5.93E-01	
bHLH	*Glyma01g02390*		1.76E-02	1.50E-01			HD-ZIP	*Glyma10g38280*		1.39E-01	4.79E-01	
bHLH	*Glyma02g15520*		1.06E-01	5.88E-01			HD-ZIP	*Glyma12g10710*		3.11E-02	6.30E-01	
bHLH	*Glyma02g15780*		3.65E-02	4.20E-01			HD-ZIP	*Glyma13g38430*		4.84E-02	7.85E-01	
bHLH	*Glyma02g29830*		6.04E-01	6.09E-01			HD-ZIP	*Glyma13g43350*		2.93E-01	1.00E+00	
bHLH	*Glyma04g33920*		NA	NA			HD-ZIP	*Glyma15g13640*		5.48E-03	2.92E-02	
bHLH	*Glyma06g20520*		NA	NA			HSF	*Glyma01g39260*		4.16E-05	8.30E-09	TRUE
bHLH	*Glyma06g41620*		2.56E-04	1.15E-01			HSF	*Glyma05g34450*	TRUE	8.19E-01	1.00E+00	
bHLH	*Glyma07g03100*		1.22E-01	8.48E-01			HSF	*Glyma09g26510*		6.97E-03	1.42E-09	TRUE
bHLH	*Glyma07g32690*		6.59E-01	1.00E+00			HSF	*Glyma13g16510*	TRUE	9.42E-11	2.66E-04	TRUE
bHLH	*Glyma07g32980*		8.14E-01	3.03E-06			HSF	*Glyma20g28870*	TRUE	5.85E-01	5.93E-01	
bHLH	*Glyma08g28010*		7.03E-02	6.27E-02			HSF	*Glyma20g29610*		5.56E-16	1.43E-77	TRUE
bHLH	*Glyma08g37240*		5.44E-02	8.60E-06			HSF	*Glyma03g34900*		6.04E-01	6.62E-01	
bHLH	*Glyma09g06770*		1.91E-01	5.93E-01			HSF	*Glyma07g09510*		4.14E-01	2.85E-01	
bHLH	*Glyma10g12150*		5.85E-01	4.67E-01			HSF	*Glyma09g32300*		1.00E+00	2.04E-01	

bHLH	*Glyma11g04690*		NA	1.00E+00			HSF	*Glyma11g06010*		7.40E-01	2.46E-01	
bHLH	*Glyma11g35480*		4.37E-01	4.53E-01			HSF	*Glyma16g32070*		6.45E-01	1.85E-04	
bHLH	*Glyma12g16560*		2.20E-01	7.71E-01			LBD	*Glyma02g27230*	TRUE	3.88E-02	2.57E-01	
bHLH	*Glyma12g32280*		2.34E-02	1.46E-03			LBD	*Glyma09g40500*		7.65E-01	1.00E+00	
bHLH	*Glyma12g34300*		8.05E-03	1.16E-01			LBD	*Glyma12g07650*		4.37E-01	4.53E-01	
bHLH	*Glyma13g27880*		5.85E-01	5.93E-01			LBD	*Glyma13g07590*		1.89E-02	8.46E-01	
bHLH	*Glyma13g36260*		1.04E-02	9.33E-01			LBD	*Glyma15g13410*		NA	NA	
bHLH	*Glyma13g39650*		NA	NA			LBD	*Glyma18g45320*		5.69E-01	2.46E-02	
bHLH	*Glyma15g00730*		1.00E+00	2.46E-01			LSD	*Glyma07g31600*		2.64E-03	1.37E-06	TRUE
bHLH	*Glyma17g09810*		1.00E+00	1.00E+00			MIKC	*Glyma02g04710*		1.00E+00	NA	
bZIP	*Glyma06g08390*		4.68E-13	3.65E-08	TRUE		MIKC	*Glyma05g28130*		6.54E-01	4.79E-01	
bZIP	*Glyma10g08370*		4.66E-21	5.97E-09	TRUE		MIKC	*Glyma05g28140*		8.12E-01	6.23E-01	
bZIP	*Glyma14g07800*		1.68E-05	4.12E-07	TRUE		MIKC	*Glyma06g02990*		1.00E+00	1.00E+00	
bZIP	*Glyma17g29170*	TRUE	1.75E-01	1.83E-01			MIKC	*Glyma08g11110*		2.50E-01	5.00E-02	
bZIP	*Glyma19g30230*		1.30E-06	1.43E-04	TRUE		MIKC	*Glyma08g12730*		1.02E-02	5.07E-01	
bZIP	*Glyma02g09140*		2.68E-01	2.12E-01			MIKC	*Glyma10g38580*		1.00E+00	5.04E-01	
bZIP	*Glyma03g00580*		3.70E-03	3.70E-02			MIKC	*Glyma20g29250*		1.91E-01	1.00E+00	
bZIP	*Glyma03g28320*		1.59E-01	8.77E-01			M-type	*Glyma08g03820*		NA	NA	
bZIP	*Glyma07g06620*		9.18E-01	6.69E-01			MYB	*Glyma04g04490*		4.18E-13	8.39E-63	TRUE
bZIP	*Glyma08g08220*		6.63E-01	1.71E-01			MYB	*Glyma07g15850*		6.74E-21	2.28E-16	TRUE
bZIP	*Glyma09g34170*		6.73E-02	5.48E-01			MYB	*Glyma16g02570*		8.15E-05	7.23E-04	TRUE
bZIP	*Glyma12g03690*		9.01E-02	3.34E-02			MYB	*Glyma02g13770*		NA	NA	
bZIP	*Glyma12g04050*		6.64E-02	6.98E-01			MYB	*Glyma02g41180*		1.00E+00	4.67E-01	
bZIP	*Glyma19g37910*		3.28E-02	1.31E-01			MYB	*Glyma03g19030*		8.02E-01	4.10E-01	
C2H2	*Glyma01g04310*	TRUE	NA	NA			MYB	*Glyma03g38040*		6.59E-01	1.00E+00	

C2H2	*Glyma03g33700*	TRUE	1.00E+00	1.00E+00			MYB	*Glyma05g04900*		1.00E+00	7.79E-01	
C2H2	*Glyma16g25550*		5.98E-05	9.35E-03	TRUE		MYB	*Glyma07g05960*		5.08E-01	6.80E-02	
C2H2	*Glyma03g31390*		NA	NA			MYB	*Glyma07g16980*		NA	NA	
C2H2	*Glyma04g36620*		1.39E-01	5.64E-01			MYB	*Glyma08g20150*		1.30E-02	1.07E-01	
C2H2	*Glyma05g04690*		1.00E+00	5.36E-01			MYB	*Glyma10g06190*		3.25E-01	6.64E-01	
C2H2	*Glyma07g27820*		8.19E-01	1.00E+00			MYB	*Glyma12g32610*		NA	NA	
C2H2	*Glyma08g14320*		5.08E-01	5.04E-01			MYB	*Glyma17g13010*		7.62E-02	6.95E-03	
C2H2	*Glyma08g16390*		4.91E-05	1.03E-01			MYB	*Glyma17g15270*		6.27E-01	4.72E-01	
C2H2	*Glyma10g40400*		5.75E-02	8.72E-07			MYB	*Glyma20g22230*		1.21E-01	4.64E-01	
C2H2	*Glyma11g03600*		1.91E-01	1.00E+00			MYB_related	*Glyma04g03400*		1.79E-13	2.37E-05	TRUE
C2H2	*Glyma12g09000*		9.10E-02	1.00E+00			MYB_related	*Glyma13g20760*	TRUE	6.34E-03	8.76E-02	
C2H2	*Glyma13g19560*		1.37E-01	3.54E-02			MYB_related	*Glyma06g10170*		3.85E-01	8.68E-01	
C2H2	*Glyma15g42870*		NA	NA			MYB_related	*Glyma12g10790*		3.15E-01	4.34E-05	
C3H	*Glyma04g36670*	TRUE	1.47E-04	5.36E-03	TRUE		MYB_related	*Glyma12g12990*		NA	NA	
C3H	*Glyma05g02920*	TRUE	4.74E-02	7.62E-01			MYB_related	*Glyma16g03640*		3.58E-01	1.00E+00	
C3H	*Glyma05g36110*		1.97E-07	3.99E-03	TRUE		MYB_related	*Glyma17g09310*		2.52E-01	7.63E-01	
C3H	*Glyma06g44440*		6.21E-31	1.29E-09	TRUE		MYB_related	*Glyma17g17370*		1.27E-02	4.18E-02	
C3H	*Glyma07g37980*	TRUE	9.69E-04	5.91E-02			MYB_related	*Glyma18g47230*		1.00E+00	1.26E-01	
C3H	*Glyma12g13300*		3.73E-34	3.10E-09	TRUE		NAC	*Glyma01g06150*	TRUE	4.37E-01	1.00E+00	
C3H	*Glyma12g33320*		8.79E-37	1.14E-07	TRUE		NAC	*Glyma02g12220*	TRUE	1.00E+00	1.00E+00	
C3H	*Glyma02g29360*		3.16E-01	5.71E-01			NAC	*Glyma02g26480*		4.17E-32	9.06E-84	TRUE
C3H	*Glyma08g16340*		6.54E-01	1.00E+00			NAC	*Glyma02g38710*	TRUE	5.56E-04	2.92E-01	
C3H	*Glyma10g44330*		7.31E-01	3.69E-02			NAC	*Glyma04g42800*		4.85E-06	4.46E-36	TRUE
C3H	*Glyma15g42750*		8.27E-02	7.07E-01			NAC	*Glyma06g11970*		1.25E-23	7.56E-67	TRUE
C3H	*Glyma18g47660*		5.17E-01	7.40E-01			NAC	*Glyma06g14290*	TRUE	2.36E-03	8.33E-05	TRUE

C3H	*Glyma20g36140*		1.83E-01	7.30E-01			NAC	*Glyma07g35630*	TRUE	4.31E-01	3.13E-07	
C3H	*Glyma20g39160*		3.59E-01	1.09E-05			NAC	*Glyma08g19300*		1.90E-04	7.78E-14	TRUE
CAMTA	*Glyma05g31190*		6.59E-01	2.46E-01			NAC	*Glyma12g35000*	TRUE	8.59E-04	4.22E-05	TRUE
CO-like	*Glyma19g27240*		1.00E+00	4.91E-02			NAC	*Glyma13g35550*	TRUE	6.46E-03	2.65E-16	TRUE
DBB	*Glyma11g07930*		2.20E-03	7.74E-03	TRUE		NAC	*Glyma14g24220*		3.13E-03	6.04E-18	TRUE
DBB	*Glyma01g37370*		6.68E-01	5.73E-01			NAC	*Glyma14g36840*	TRUE	1.19E-05	6.77E-02	
DBB	*Glyma09g14880*		7.42E-02	5.73E-01			NAC	*Glyma20g33390*	TRUE	1.13E-01	3.34E-02	
DBB	*Glyma12g05570*		1.97E-01	4.83E-02			NAC	*Glyma04g38560*		3.34E-01	8.23E-13	
DBB	*Glyma12g36260*		5.94E-01	7.15E-01			NAC	*Glyma05g24910*		NA	1.00E+00	
Dof	*Glyma16g26030*	TRUE	4.12E-01	1.33E-01			NAC	*Glyma07g31220*		1.00E+00	1.00E+00	
Dof	*Glyma19g38660*	TRUE	4.37E-01	4.53E-01			NAC	*Glyma08g08010*		1.00E+00	1.00E+00	
Dof	*Glyma19g38750*	TRUE	NA	NA			NAC	*Glyma08g17140*		1.00E+00	5.04E-01	
Dof	*Glyma13g31110*		8.35E-02	6.64E-01			NAC	*Glyma14g09240*		5.58E-02	1.21E-01	
Dof	*Glyma18g38560*		1.46E-01	5.09E-02			NAC	*Glyma15g05690*		4.37E-01	4.53E-01	
E2F/DP	*Glyma12g09860*		1.39E-01	4.79E-01			NAC	*Glyma15g07620*		1.00E+00	1.00E+00	
E2F/DP	*Glyma17g10130*		7.97E-02	1.97E-01			NAC	*Glyma19g08510*		5.58E-02	6.09E-02	
EIL	*Glyma05g31410*	TRUE	1.00E+00	1.68E-02			NF-YA	*Glyma12g36540*		8.50E-20	1.72E-11	TRUE
EIL	*Glyma18g02190*	TRUE	4.51E-01	2.04E-09			NF-YB	*Glyma09g01650*		9.71E-04	1.32E-03	TRUE
ERF	*Glyma02g42960*		2.32E-08	1.33E-25	TRUE		NF-YB	*Glyma15g12570*		2.44E-08	9.66E-07	TRUE
ERF	*Glyma03g41640*	TRUE	NA	NA			NF-YB	*Glyma20g00240*		2.71E-04	9.15E-04	TRUE
ERF	*Glyma04g39510*	TRUE	1.05E-12	5.16E-13	TRUE		NF-YB	*Glyma05g07750*		1.86E-02	5.34E-01	
ERF	*Glyma05g19050*		1.79E-05	2.12E-05	TRUE		NF-YB	*Glyma11g18960*		9.18E-01	2.74E-02	
ERF	*Glyma05g31370*	TRUE	2.35E-01	2.97E-05			NF-YC	*Glyma06g17780*	TRUE	4.97E-03	6.17E-01	
ERF	*Glyma05g32040*	TRUE	4.79E-01	4.46E-05			NF-YC	*Glyma15g36170*	TRUE	1.96E-02	1.39E-01	
ERF	*Glyma06g03110*	TRUE	1.41E-28	4.92E-08	TRUE		NF-YC	*Glyma08g17630*		3.52E-01	1.77E-02	

ERF	*Glyma06g08990*	TRUE	5.34E-05	6.49E-01			NF-YC	*Glyma11g20740*		7.25E-01	4.25E-01	
ERF	*Glyma08g02460*	TRUE	2.40E-03	8.01E-10	TRUE		NF-YC	*Glyma13g27790*		2.62E-01	NA	
ERF	*Glyma08g15350*	TRUE	3.00E-06	6.42E-12	TRUE		S1Fa-like	*Glyma03g33640*		1.35E-06	3.62E-02	
ERF	*Glyma09g04630*	TRUE	2.66E-42	1.78E-04	TRUE		SBP	*Glyma13g24590*	TRUE	NA	NA	
ERF	*Glyma10g36760*		7.00E-03	8.56E-04	TRUE		SRS	*Glyma13g26730*	TRUE	2.52E-01	4.81E-01	
ERF	*Glyma14g06080*		6.70E-08	9.05E-17	TRUE		SRS	*Glyma15g37600*	TRUE	1.00E+00	1.00E+00	
ERF	*Glyma14g06290*		5.02E-16	1.08E-10	TRUE		SRS	*Glyma02g05830*		1.00E+00	1.00E+00	
ERF	*Glyma14g07620*		1.66E-32	7.58E-10	TRUE		SRS	*Glyma04g03000*		1.00E+00	1.00E+00	
ERF	*Glyma14g09320*		4.41E-04	5.48E-21	TRUE		SRS	*Glyma14g03900*		3.34E-03	1.22E-01	
ERF	*Glyma15g16260*	TRUE	3.20E-103	9.40E-253	TRUE		TALE	*Glyma02g06730*	TRUE	7.41E-05	3.63E-02	
ERF	*Glyma16g27950*		4.92E-03	2.09E-10	TRUE		TALE	*Glyma04g06810*		6.32E-04	9.37E-02	
ERF	*Glyma17g31900*	TRUE	1.00E+00	2.57E-01			TCP	*Glyma03g02090*	TRUE	4.20E-01	8.22E-01	
ERF	*Glyma17g35860*		5.41E-12	2.63E-13	TRUE		TCP	*Glyma13g29160*	TRUE	4.39E-01	4.84E-01	
ERF	*Glyma20g16920*	TRUE	5.57E-01	1.00E+00			Trihelix	*Glyma03g34730*		1.83E-17	2.58E-15	TRUE
ERF	*Glyma01g39540*		1.06E-01	1.09E-05			Trihelix	*Glyma09g38050*	TRUE	5.03E-01	2.36E-12	
ERF	*Glyma02g31350*		5.08E-01	5.04E-01			Trihelix	*Glyma10g07730*	TRUE	2.52E-01	2.57E-01	
ERF	*Glyma03g26530*		4.37E-01	4.53E-01			Trihelix	*Glyma07g18320*		2.52E-01	1.00E+00	
ERF	*Glyma03g42450*		1.69E-05	2.17E-02			Trihelix	*Glyma10g36960*		3.49E-02	8.91E-03	
ERF	*Glyma06g45680*		1.80E-02	6.92E-02			Trihelix	*Glyma13g26550*		4.70E-01	1.11E-01	
ERF	*Glyma07g37410*		NA	NA			Trihelix	*Glyma18g51790*		4.40E-01	3.19E-01	
ERF	*Glyma09g05850*		NA	NA			WOX	*Glyma13g41000*		NA	NA	
ERF	*Glyma09g05860*		NA	NA			WRKY	*Glyma13g00380*		5.80E-05	3.87E-11	TRUE
ERF	*Glyma10g04210*		NA	NA			WRKY	*Glyma15g11680*		4.48E-09	1.19E-28	TRUE
ERF	*Glyma11g02050*		NA	NA			WRKY	*Glyma19g02440*	TRUE	NA	1.00E+00	
ERF	*Glyma12g32400*		5.73E-02	6.79E-02			WRKY	*Glyma02g46280*		1.00E+00	1.00E+00	

ERF	*Glyma13g29920*		NA	NA			WRKY	*Glyma05g31800*		NA	NA	
ERF	*Glyma13g38030*		6.22E-03	3.95E-02			WRKY	*Glyma08g08340*		4.37E-01	4.53E-01	
ERF	*Glyma13g39540*		NA	1.00E+00			WRKY	*Glyma08g15050*		4.67E-02	7.36E-01	
ERF	*Glyma15g00660*		NA	5.04E-01			WRKY	*Glyma08g43770*		1.10E-01	2.39E-01	
ERF	*Glyma15g08560*		NA	NA			WRKY	*Glyma09g00820*		1.50E-01	2.55E-04	
ERF	*Glyma15g09190*		4.37E-01	1.00E+00			WRKY	*Glyma09g03900*		1.00E+00	4.53E-01	
ERF	*Glyma16g04410*		NA	5.04E-01			WRKY	*Glyma10g37460*		NA	1.00E+00	
ERF	*Glyma16g05190*		NA	NA			WRKY	*Glyma13g38630*		3.95E-01	7.99E-02	
ERF	*Glyma16g26320*		2.24E-02	2.61E-02			WRKY	*Glyma16g05880*		NA	NA	
ERF	*Glyma17g13320*		4.37E-01	1.00E+00			WRKY	*Glyma18g09040*		1.73E-02	3.01E-01	
ERF	*Glyma18g33460*		7.02E-01	1.26E-01			WRKY	*Glyma19g26400*		7.25E-02	1.00E+00	
ERF	*Glyma19g29000*		NA	1.00E+00			WRKY	*Glyma19g40950*		5.85E-01	5.93E-01	
FAR1	*Glyma15g03440*	TRUE	7.07E-01	7.09E-01			ZF-HD	*Glyma05g01060*		1.00E+00	1.00E+00	
G2-like	*Glyma10g04540*		6.27E-04	1.93E-03	TRUE		ZF-HD	*Glyma06g09970*		7.25E-01	3.45E-09	
G2-like	*Glyma13g37010*		3.46E-08	2.64E-06	TRUE		ZF-HD	*Glyma13g05380*		6.50E-03	4.15E-02	
G2-like	*Glyma17g36500*	TRUE	9.10E-02	1.84E-01			ZF-HD	*Glyma17g00590*		1.69E-01	2.39E-01	
G2-like	*Glyma04g21680*		1.00E+00	1.00E+00			ZF-HD	*Glyma20g18460*		2.62E-01	5.04E-01	
G2-like	*Glyma10g42750*		3.95E-01	2.31E-01			ZF-HD	*Glyma20g18520*		1.37E-01	1.00E+00	
G2-like	*Glyma12g33430*		2.62E-01	3.54E-02								

Among all the 327 predicted TFs, 61 were differentially expressed in high and low-oil soybean accessions and 67 were high-oil dicot-specific. Group H-L1: high-oil HD5 and low-oil ZYD4364; Group H-L2: HD5 and low-oil Y117249. Genes that were dicot-specific or significantly differentially expressed in the two groups were indicated by “TRUE”.
